# Cultural proximity reveals a local premium in collective memory

**DOI:** 10.1093/pnasnexus/pgag308

**Published:** 2026-09-21

**Authors:** Mariana Macedo, Melanie Oyarzun, Cristian Candia, César A Hidalgo

**Affiliations:** School of Computer Science and Engineering, Constructor University, Bremen 28759, Germany; Center for Collective Learning, CIAS, Corvinus University of Budapest, Budapest 1093, Hungary; Instituto de Data Science, Facultad de Ingeniería, Universidad del Desarrollo, Las Condes 7610658, Chile; Northwestern Institute on Complex Systems (NICO), Northwestern University, Evanston, IL 60208, USA; Center for Collective Learning, CIAS, Corvinus University of Budapest, Budapest 1093, Hungary; Center for Collective Learning, IAST, Toulouse School of Economics, Université de Toulouse Capitole, Toulouse 31080, France; Alliance Manchester Business School, University of Manchester, Manchester M15 6PB, United Kingdom

## Abstract

Collective memory—shared information about the past that shapes a group's identity—is often examined through constructs such as familiarity or salience, conflating its local and global components. Here, we untangle these components using multilingual online attention traces for over 50,000 Wikipedia biographies spanning 137 billion pageviews and a large-scale behavioral recognition experiment in which more than 41,000 participants answered factual questions about famous biographies. Our experiment reveals a robust local memory premium, with people being around 10% points more likely to answer correctly questions about someone from their country and about five points more likely to answer correctly questions about biographies with whom they share a language. Finally, we study the impact of three major life events: Nobel Prizes, Oscars, and deaths in local and global online attention, finding these events produce brief but strong surges in local attention and slower but sustained increases in global attention. These findings add important spatial and temporal nuances to our understanding of collective memory.

Significance Statement Collective memory shapes how societies make sense of the past, yet its measurement often ignores geographic and linguistic structure. Here, we use Wikipedia attention traces and an online recognition experiment played by over 40,000 volunteers to estimate local and global collective memory. We find a robust local attention premium of roughly 10% points for subjects answering questions about biographies from their country and about 5% points for biographies of people who share their language. Finally, we show that major life events produce distinct trajectories, with brief surges in local attention and slower, sustained growth in global attention. These findings offer a spatially aware framework for understanding the local and global dimensions of collective memory.

## Introduction

Bernardo O’Higgins is well known in Chile but not elsewhere in the world. O’Higgins was a military leader during Chile's independence war. A founding father who left a vast legacy in the long country sitting on the southwest of the Andes. Today, it is hard to find a place in Chile without a major street or avenue named after O’Higgins. But while the name O’Higgins is as recognizable to Chileans as the name Napoleon is to the French, the latter is also recognized throughout much of the world.

O’Higgins' local fame and relative global obscurity are an example of how collective memory can vary between local and global contexts. While not particularly controversial, this disparity raises questions about the diffusion and preservation of local and global forms of collective memory.

Collective memory—the shared information about the past that shapes group identity—plays a central role in how societies remember and forget. Yet, empirical studies of collective memory often rely on a single, underspecified construct—typically labeled familiarity, salience, or notability—to quantify who is remembered. These constructs serve as broad proxies for remembrance but conflate global fame and local prominence. As a result, it remains unclear whether collective memory reflects exposure to widely visible figures, saturation within local contexts, or identification with culturally proximate individuals. Disentangling these components of collective memory is essential for understanding how different forms of attention and identity jointly shape what societies remember and forget.

In recent years, several studies have explored the structure of collective memory using data on paper and patent citations and visits to websites such as Wikipedia (1–11). Ronen et al. (9) studied the impact of language networks on the online popularity of biographies, showing that attention correlates with the centrality of a language in three networks of bilingual expressions. Jara-Figueroa et al. (12) explored how the introduction of new technologies is followed by changes in the volume and composition of biographical records. For example, prior to printing, they found relatively few biographies of famous scientists and artists, just as there were few biographies of performing artists—such as singers and actors—before the introduction of film, radio, and television. Schich et al. (13) used biographical data to estimate a network of historical migration in an effort to understand the dynamics of cultural centers, and Koch et al. used similar migration data to study the historical impact of migrants on the cultural diversification of European cities (14) and to estimate the historical GDP per capita of regions in the United States and Europe (15).

Besides exploring the impact of languages, technology, and migration on collective memory, researchers have also studied the universality of its temporal decay (1, 3, 10, 11, 16, 17) and the attention “bumps” triggered by tragic events, such as airplane crashes (18) or death (19). Collective memory is known to decay following a universal two-step function (3, 10, 19, 20), starting with a fast and short-lived decay attributed to communicative memory, followed by a slower and longer-lived decay attributed to cultural memory. Death also impacts collective memory, resulting in an attention boost, especially when these deaths are unnatural or occur at a young age (19).

Yet, despite this progress, our quantitative understanding of collective memory still suffers from important limitations. One of them is that—for the most part—these studies tend to focus on estimates of collective memory that fail to distinguish between local and global forms of attention.

Compare Johnny Unitas and Elinor Ostrom. Both were born in the United States in 1933. Unitas was an American football player who won four national championships and was named three times the most valuable player of the league. Ostrom was a political scientist whose influential work on common pool resource problems made her the first woman to earn a Nobel Prize in Economics. In recent years, Unitas' Wikipedia page received between 19,000 and 48,000 monthly views, most of them (more than 80%) in English. By contrast, Ostrom's page received fewer views in total (between 14,000 and 23,000 a month), but a relatively higher proportion in non-English languages, with only 55 to 65% of these views in English (Fig. 1). This shows that, while Unitas and Ostrom are both highly accomplished, their patterns of attention are different. Unitas is more “local,” receiving little attention outside the English-speaking world, whereas Ostrom's legacy seems more global, with her impact transcending geographic, linguistic, and cultural barriers.

**Figure 1:**
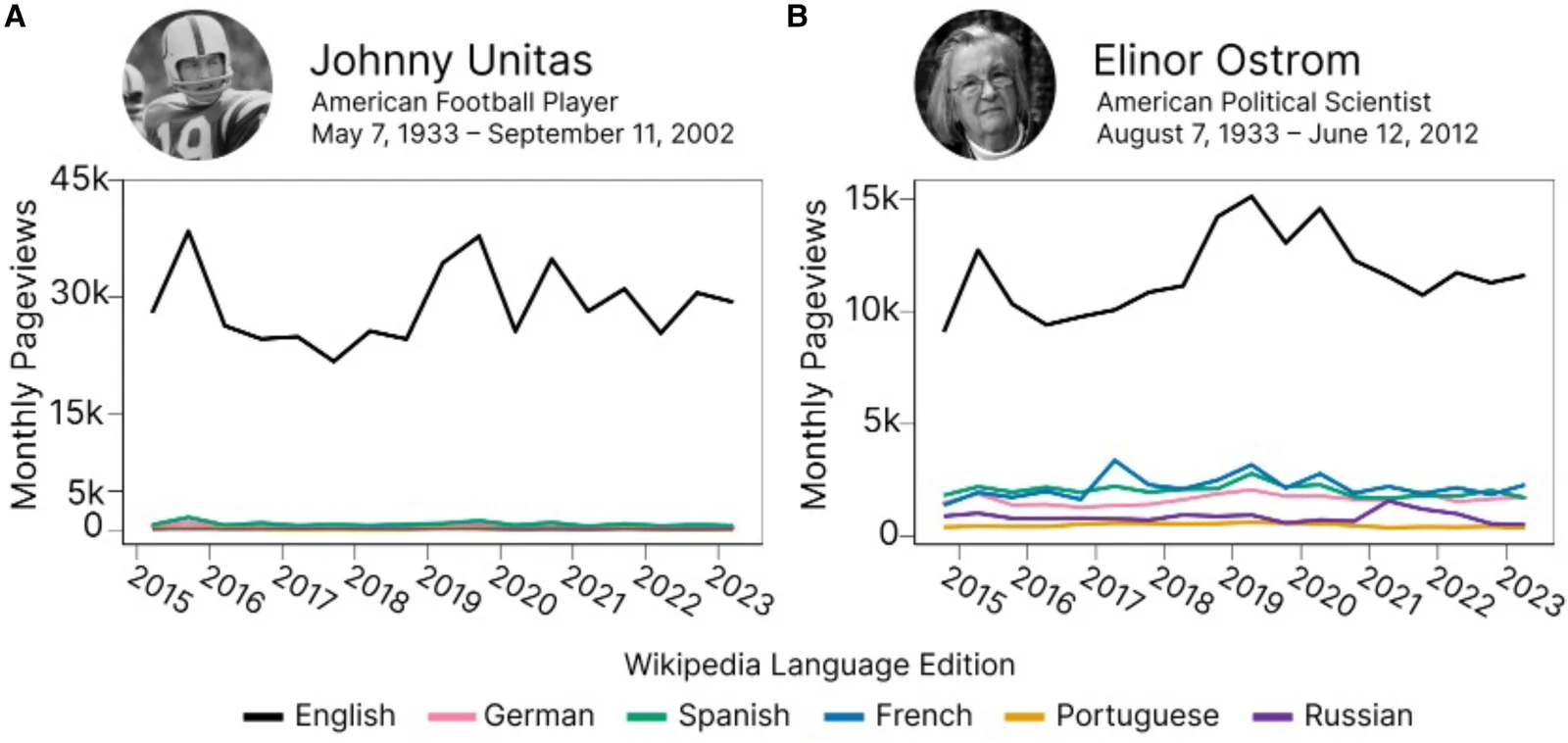
Pageviews for the Wikipedia biographies of A) Johnny Unitas and B) Elinor Ostrom in their six most visited languages. Image credit: Johnny Unitas' image is in the public domain. Elinor Ostrom's portrait is by © Holger Motzkau 2010, Wikipedia/Wikimedia Commons (CC BY-SA 3.0).

We use this example to motivate two dimensions of collective memory: a local and a global component, which we can unpack using data on the distribution of online attention received by biographies across the different language editions of Wikipedia. However, since Wikipedia attention suffers from self-selection, we need to validate these measures using data involving a source of external variation. Here, we draw on data we collected through an online trivia game where more than 41,000 players answered questions about historical figures with different levels of local and global fame. In the game, players did not choose the biographies, providing exogenous variation that required players to answer questions about biographies who might or might not share a place of origin and/or language with them. We find that our measures of online attention positively correlate with the probability that a player answered a question about a biography correctly, validating them as proxy measures of collective memory.

Additionally, we use the data from the experiment to estimate premia for shared geography and language. We find that players show higher recognition, a higher probability of answering correctly questions about biographies of people with whom they share a country or a language.

Finally, we examine how the local and global attention received by these Wikipedia biographies changes with three key events: receiving an Oscar, a Nobel, and death. We find that these events result in an intense but short-lived spike in local attention and a modest but sustained boost in global attention.

Together, these findings contribute to our understanding of the dynamics and structure of local and global forms of collective memory.

## Results

We begin by defining our estimates of local and global collective memory. Following previous studies (3, 5, 7, 12), we proxy online attention using data from Wikipedia, drawing on 158,170 biographies from Pantheon.world (5) and 2,268,329 from Laouenan et al. (7), both with available birth date and occupation information. For each biography, we extracted monthly pageviews in every language edition in which it appeared, covering the period from 2015 to 2024 using the Wikimedia API. We excluded biographies that had any month without pageviews or fewer than 1,000 total pageviews between 2020 and 2022. These filters resulted in a final sample of 51,465 biographies, including 27,705 biographies of individuals who were still alive in 2024.

Figure 2A shows the vectors representing the pageviews Preceived respectively by the biographies of Johnny Unitas and Elinor Ostrom in 2022 across six different Wikipedia language editions. We use these vectors to illustrate our two metrics.

**Figure 2:**
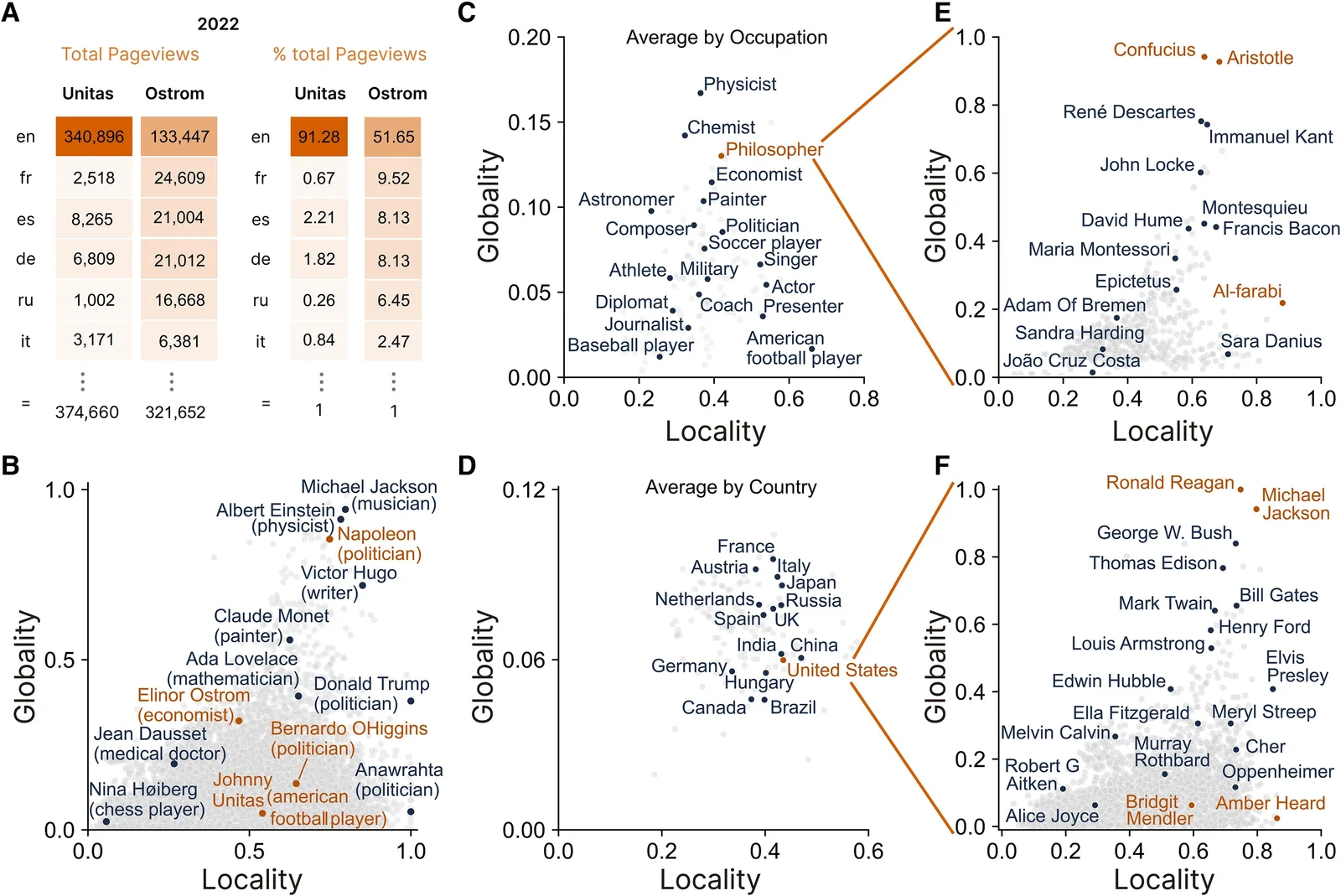
Unpacking local and global collective memory. A) Number and percentage of Wikipedia pageviews received by the biographies of Johnny Unitas and Elinor Ostrom in 2022 across six selected languages (English: En, French: Fr, Spanish: Es, German: De, Russian: Ru, Italian: It). We compute locality and globality from the full version of this vector (including all languages in which Wikipedia is available). In Fig. S2, we replicate this illustration considering dead and alive biographies separately. B) Scatter plot of locality and globality with a few selected biographies. C) Average locality and globality by occupation. D) Average locality and globality by country of birth. E) Locality and globality of the biographies of philosophers. F) Locality and globality of biographies for individuals born in the United States.

First, we define the *locality*  $C_{i}$. of a biography. For each Wikipedia language edition *l*, we compute the ratio of its pageviews $P_{il}$ to those of the most-viewed biography in that language, $\underset{j}{\max}\;P_{\;jl}$. The locality of biography *i* is defined as the highest of these ratios across all languages:


(1) $$C_{i}=\mathrm{MinMax}\;\underset{l}{\max}\;\left(\frac{\log\;(P_{il\;})}{\log\;(\;\underset{\;j\;\in \;I}{\max}\;(P_{\;jl\;}))}\right).$$

We apply a logarithmic transformation to compress the extreme values and expand the lower ones, producing a more balanced distribution for our metric, and we add a Min–Max normalization to map the values between 0 and 1, preserving the shape of the distribution. Thus, locality $C_{i}$ reaches its maximum value of 1 when a biography *i* receives the largest number of pageviews in one of the languages in our dataset. In the case of Unitas and Ostrom, their respective values for locality *C* in 2022 are *C*_Unitas_ = 0.57 and *C*_Ostrom_ = 0.49, with their maximums in English.

For our global metric, we compute the percentage of languages in which a biography has an above-average presence. We do this using a specialization indicator known as revealed comparative advantage RCA (21, 22)—the ratio between the observed and expected number of pageviews. Formally, we let the RCA of biography *i* for each language *l*. in a given time period be:


(2) $$R_{il}=\frac{P_{il}P}{P_{i}P_{l}},$$

where omitted indexes have been added (e.g. $P_{i}\;=\;\underset{l}{\sum }\;P_{il}$)Using this definition, we can define the *globality G_i_* of biography *i* for a given time period as the normalized proportion of languages in which its pageviews exceed the expected value:


(3) $$G_{i}=\mathrm{MinMax}\left(\frac{count(R_{i}>1)}{N_{l}}\right),$$

where $N_{l}$ is the total number of languages in our dataset. We also apply Min–Max normalization to map all the values between 0 and 1, maintaining the shape of the distribution. In the case of Unitas and Ostrom, we obtain values of *G*_Unitas_ = 0.049 and *G*_Ostrom_ = 0.32.

Locality (*C*) and globality (*G*) allow us to decompose the online attention received by biographies (Fig. 2B). As we expected, Ostrom is “more global” but “less local” than Unitas, while Napoleon is substantially “more global” than O’Higgins and only slightly “more local.” Figure 2B also shows that locality and globality in 2022 are weakly correlated (*R*^2^ = 5.8%, Pearson = 0.24, Spearman = 0.178), indicating that while these are two different dimensions, they are not fully independent. In fact, biographies that score low in locality also tend to score low in globality, particularly within the lower end of the locality distribution (e.g. *R*^2^ = 14.9%, Pearson = 0.386, Spearman = 0.4339 when *C* < 0.2). At high levels of locality, this correlation weakens (*R*^2^ = 0.30%, Pearson = 0.055, Spearman = −0.009 when *C* > 0.8) since some biographies with a strong local presence are not necessarily global. Thus, at lower levels of recognition, locality and globality tend to go hand in hand, but at higher levels of recognition, biographies can be markedly local without necessarily being global.

Next, we aggregate globality and locality scores by occupations (Fig. 2C) and country of birth (Fig. 2D), finding substantial heterogeneity across occupations and countries. Some occupations, such as philosophers and physicists, have—on average—higher indicators of globality than others, such as Olympic athletes and American football players. Figure 2E decomposes this heterogeneity again among Philosophers, showing that some philosophers such as Confucius and Aristotle are remembered across the world, while others like Al-Farabi are remembered prominently in a few languages (e.g. Kazakh and Turkish).

Figure 2D groups biographies by country of birth. Here, France and Italy rank among the highest in globality, with a large share of prominent physicists, philosophers, and painters (France: 14.45% physicists, 17.93% philosophers, and 18.89% painters; and Italy: 4.94, 8.42, and 20.31%, respectively). Yet, the average birth year of global biographies from France (1821) and Italy (1769) suggests a more historical rooted source of influence (Table S3). Countries such as Brazil score lower in globality, being the birthplace of few physicists, philosophers, and painters (0.55, 0.88, and 0.43%, respectively) and of many soccer players (6.80%), resulting in a globality of more recent vintage (average birth year of 1963). The United States, the largest contributor to the dataset (10,774 biographies (21.7%), Table S1 for details), is a more peculiar case. It spans the full spectrum of occupations and has a relatively recent vintage for global biographies (average year of birth 1925) (Fig. 2F), yet it also has a large number of locally famous figures, reducing its average globality.

Next, we compare our estimates of locality and globality with experimental data collected through an online trivia game deployed on a website dedicated to collective memory (https://pantheon.world). The experimental protocol was approved by the internal review board of the Toulouse School of Economics IAST (NUMBER 2022-07-001). Further details about the experimental setup are available in the Methods section and the Supplementary material.

In the game, players answered multiple-choice questions about famous historical figures. Each question offered four possible answers generated programmatically following simple templates such as: “John Lennon was a: (a) musician, (b) astronaut, (c) sociologist, or (d) biologist” (see Fig. 3A). In this example, the correct answer is “(a) musician.” Going forward, we interpret the fraction of correct answers as a measure of players' recognition of basic biographical facts about a historical figure. To deter players from searching for the answers online, we gave them only 15 seconds to respond to each question (after which the game moves on to the next question automatically). Questions were also updated daily to encourage repeated participation. To collect demographic information, we asked players to voluntarily complete a demographic survey (age, nationality, gender, and languages spoken) after their first game. The following results come from a sample of 41,070 players who provided 414,290 answers between November 2022 and April 2024.

**Figure 3:**
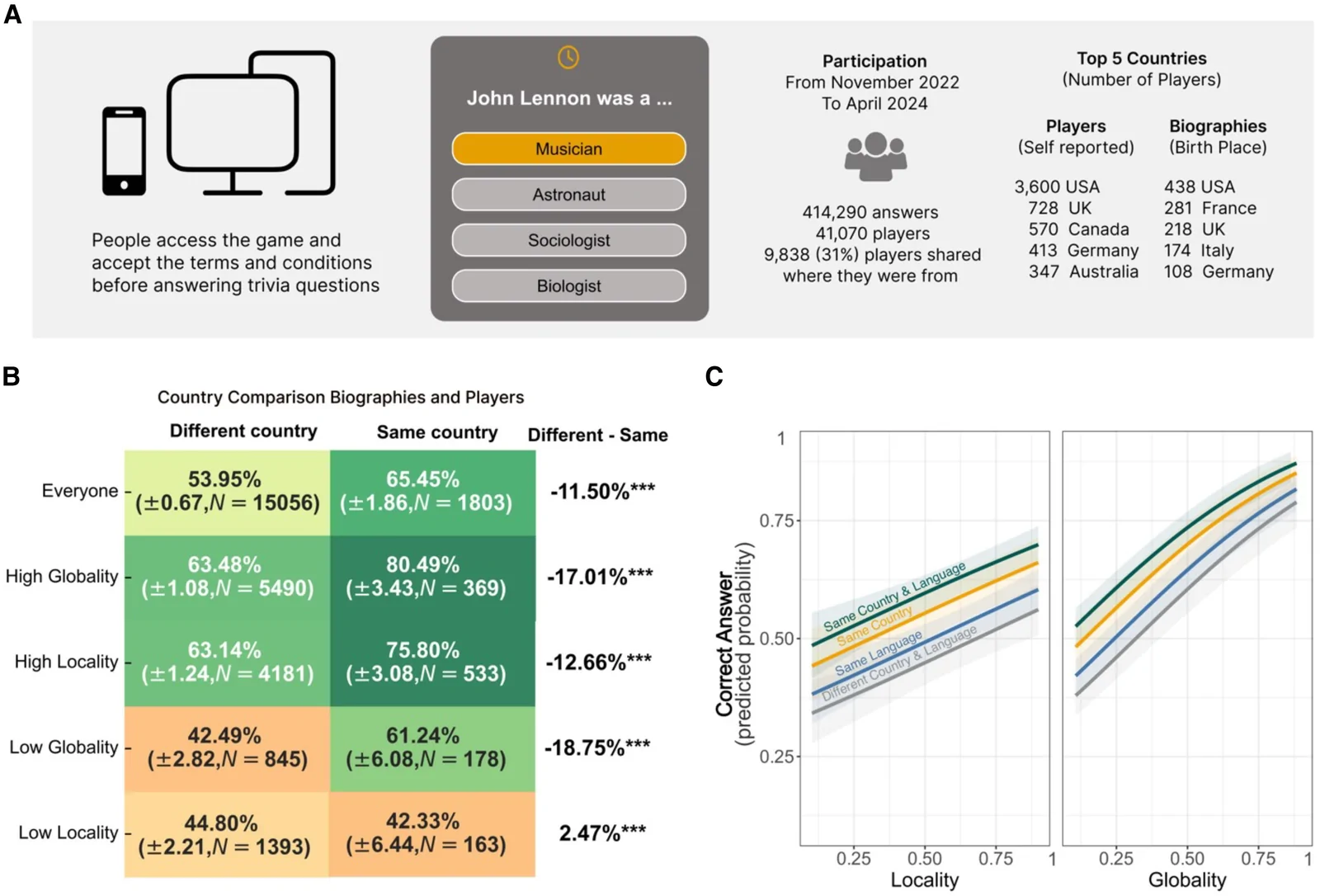
Experimentally testing the impact of shared countries and languages in collective memory. A) Schematic of the trivia game used to estimate recall rates for different biographies and basic statistics of the data collected. In total, we collected 414,290 responses from 41,070 players. B) Percentage of correct answers in the trivia game grouped by: different country and same country (columns) and by characteristics of the biographies (rows): Everyone (all biographies), high and low globality and locality (defined as the first and last quintiles of the distribution). C) Predicted probability of correct answers from the logistic model (Eq. 4), considering player's birthplace, historical figure's attention, and controls for occupation, birth year, and other factors.

The purpose of the game was to use recognition rates to explore the collective memory of local or foreign figures. For example, we expect a player from Chile to be more likely to answer correctly a question about Bernardo O’Higgins than a player from Vietnam. This approach contrasts with relying on observational data from Wikipedia, where web traffic to a biography is self-selected. In our experiment, players do not choose the questions they answer, since each day the game provides ten questions that include biographies that might be local or foreign to the player. This exogenous source of variation provides a fairer comparison of recognition rates for local and foreign biographies than measures of web traffic.

We should note that a biography can be local to a player for different reasons, such as sharing a nationality, common cultural history (e.g. shared colonial past, religion), or common language. Our experiment collected data on two indicators of proximity between players and historical figures: shared country and shared language (details available in the Supplementary material). The shared country indicator compares the player's response to the question “Where are you from?” with each figure's country of birth. The shared language indicator compares the list of languages spoken by the player with the official languages spoken in the biography's country of birth. These two indicators help separate the geographic story, where collective memory is concentrated among those who share a country, from the linguistic story, where collective memory is concentrated among those who share a language.

To explore how recognition varies with similarity, we first examine the average accuracy of trivia responses across different groups. Figure 3B shows the descriptive results; further details are available in the Experimental data section of the Supplementary material. In these estimates, players are 11.5% points more likely to correctly answer questions about biographies from their own country (65.45%) than from foreign countries (53.95%). Players are also significantly more likely to answer questions correctly when they share a language with the biography (62.35% vs. 52.86%), a 9.49% point difference (*P* < 0.001). When we further distinguish biographies by their globality and locality scores, local figures with high scores on both dimensions (in the top quintile) and those with low globality both achieve higher recognition rates than foreign figures with comparable scores.

This pattern persists in statistical models (Eq. 4 in Materials and methods) designed to estimate how locality and globality, as well as common nationality and language, affect the probability of a correct answer while controlling for additional factors. For players, we control for their age, nationality and number of spoken languages, and for historical figures, we control for their year of birth, country of birth, and occupation.

Figure 3C shows the estimated probabilities of a correct answer for players based on whether they share a country and/or language with a historical figure. The probability of a correct response is highest when players share the biography's country and language (green), followed by sharing only the country (yellow), only the language (blue), and neither (gray). Across all groups, the probability of a correct answer increases with locality and globality. At any given level of these scores, players perform best on questions about figures with whom they share both a country and language, followed by sharing only a country, only a language, and neither. Table 1 reports estimated coefficients and the average marginal effects (AMEs) across nested specifications.

**Table 1:** Logit model estimates for the likelihood that a player answers correctly a question about a historical figure.

	(1)	(2)	(3)	(4)	(5)	(6)
**A: Log-odds of correct answer**						
Same country				0.467^c^		0.421^c^
				(0.067)		(0.069)
Same language					0.287^c^	0.175^a^
					(0.067)	(0.069)
Locality	2.769^c^		−0.006	−0.114	−0.123	−0.113
	(0.105)		(0.176)	(0.304)	(0.304)	(0.304)
Globality		3.099^c^	0.181	−0.365	−0.403	−0.354
		(0.083)	(0.463)	(0.748)	(0.748)	(0.748)
Interaction: globality × locality			4.092^c^	4.713^c^	4.744^c^	4.693^c^
			(0.708)	(1.132)	(1.131)	(1.132)
Player aged older than 40				0.552^c^	0.54^c^	0.549c
				(0.039)	(0.039)	(0.039)
(Intercept)	−1.43^c^	−1.152^b^	−0.949^a^	−1.245	−1.254	−1.268
	(0.433)	(0.424)	(0.43)	(0.695)	(0.703)	(0.703)
**B: Average marginal effects**						
Same country				0.101^c^		0.092^c^
				(0.014)		(0.015)
Same language					0.064^c^	0.039^a^
					(0.015)	(0.015)
Locality	0.663^c^		0.222^c^	0.235^c^	0.235^c^	0.234^c^
	(0.024)		(0.030)	(0.049)	(0.049)	(0.049)
Globality		0.727^c^	0.581^c^	0.510^c^	0.507^c^	0.510^c^
		(0.018)	(0.026)	(0.040)	(0.040)	(0.040)
Player older than 40				0.123^c^	0.121^c^	0.123^c^
				(0.009)	(0.009)	(0.009)
Num.Obs.	40,636	40,636	40,636	15,452	15,452	15,452
AIC	54,765.5	53,871.5	53,807.6	19,869.4	19,901.4	19,866.1
BIC	55,566.5	54,672.4	54,625.8	21,283.8	21,323.4	21,295.8
Log.Lik.	−27,289.77	−26,842.75	−26,808.8	−9,749.678	−9,764.684	−9,746.031
RMSE	0.49	0.48	0.48	0.47	0.47	0.47
Occupation FE	Yes	Yes	Yes	Yes	Yes	Yes
Birthplace FE	Yes	Yes	Yes	Yes	Yes	Yes
Birth year control	Yes	Yes	Yes	Yes	Yes	Yes
Player N lang control	No	No	No	No	Yes	Yes
Player nationality FE	No	No	No	Yes	Yes	Yes

Each column reports a different specification including a subset of the explanatory variables. Panel A displays the logit coefficients (indicating changes in log-odds) and SE in parentheses. Panel B reports the estimated AMEs for the main variables, computed by averaging observation-level marginal effects over each model's estimation sample. For the same country, we show the difference in predicted probabilities between participants who share the figure's country of birth and those who do not. For Same Language, we show the difference in predicted probabilities between participants who share a language with the historical figure and those who do not. For globality and locality, we indicate the average change in the probability of a correct response per one-unit increase in these scores (d*Y*/d*X*). Num. Obs. counts answer-level observations; sample sizes vary with the availability of participant country and language information. Significance levels: ^a^*P* < 0.05, ^b^*P* < 0.01, ^c^*P* < 0.001.

Sharing a country of birth increases the probability of a correct response by about 9 to 10% points (*P* < 0.001) (columns 6 and 4), even after accounting for the figure's birth year, occupation, and the player's nationality, as well as the globality and locality metrics. Sharing a language raises the probability of a correct answer by about 3.9 to 6.4% points (*P* < 0.001) (columns 6 and 5), using the same controls. The lower estimates occur when shared country and shared language are included together in the model, since these two variables are correlated (*r* = 0.35), a fact that attenuates but does not eliminate the effects (to 9.2% points for shared country and 3.9% points for shared language). The modest reduction for shared country and more pronounced reduction for shared language suggest that geographic and cultural proximity jointly enhance the probability of a correct answer, with shared language providing an additional, yet smaller, pathway.

There are also interesting demographic differences, although we do not have an exogenous source of variation to identify these effects (e.g. our sample of older players might not be comparable to that of younger players). That said, players older than 40 were 12.3% points (*P* < 0.001) more likely to answer a question correctly than younger players. We also observe a small difference in gender, with men having a 2.4%-point (*P* < 0.01) higher probability of answering correctly compared to those who self-reported as women or other. We note that our sample is not gender balanced. Of those who provided gender information, 65.6% identified as men, 24.1% as women, and 10.2% as other (see Table S6).

When it comes to the Wikipedia-derived estimates of locality and globality, we find that both correlate positively with recognition rates after including all controls, supporting their use as proxies of collective memory. Because these scores are scaled to the [0,1] range, the marginal effects correspond to a full-range shift: moving from the minimum to the maximum of locality is associated with an increase of about 23.3% points in the probability of a correct answer, whereas the analogous shift in globality is associated with an increase of about 51% points. Their joint effect, captured by an interaction term, is significant across all specifications, indicating that the variables are more complements than substitutes for collective memory.

To illustrate the magnitude of these effects, consider a scenario in which a player shares birthplace and language with a biography. The model predicts a 61.6% probability of answering correctly when locality, globality, and other controls are held at their average values. Increasing locality by 1 SD raises the predicted probability to 65.0%, while a 1-SD increase in globality raises it to 70.5%. Increasing both metrics by 1 SD simultaneously yields a predicted probability of 75.4% (see Table S8).

To examine how recognition of basic biographical facts changes with historical age, we grouped responses by the number of years since each figure's date of birth. We observe that accuracy declines over time, but the rate and shape of this decline differ depending on whether the figure shares the player's country. Same-country figures follow a two-phase decay pattern—an initial steep drop followed by a slower decline—whereas different-country figures exhibit a more gradual, single-phase decay. This pattern aligns with prior descriptions of communicative and cultural memory, where memory initially spreads through social interactions and later persists more diffusely (1, 3, 10, 16, 17). The estimated transition point between phases occurs later for same-country figures (∼131 years) than for different-country ones (around 64 years), suggesting longer persistence of socially mediated recognition for local figures. A detailed analysis and visualization are provided in the Supplementary material (Temporal decay of collective memory section).

Lastly, we explore how significant events—such as receiving major awards (e.g. Oscars, Nobel Prizes) or death—shape the dynamics of collective memory. Using a time-event framework (23, 24) described in Eq. 5 (Materials and methods), we quantify changes in overall, local, and global attention using Wikipedia pageview data from 2015 onward. Attention to each biography is measured over time and relative to its baseline level 1 year before the event. By including biography and year fixed effects, the model accounts for stable differences across figures and for temporal shocks that affect all biographies in a given year. This approach allows us to link changes in attention more directly to the timing of the event itself, rather than to pre-existing differences across figures or global trends.

We observe distinct temporal patterns in Fig. 4 for the three types of events happening between 2015 and 2024: deaths (Fig. 4A–C), Oscars for best actor, best actress, best supporting actor, best supporting actress, best director, and best original screenplay (Fig. 4D–F) and Nobel Prizes across all fields (Fig. 4G–I). We find that death is followed by a prominent but short-lived spike in pageviews (Fig. 4A) (an increase of 181.1% (*P* < 0.001)) that declines to 17% after 1 year (*P* < 0.001), to 6.5% after 2 years (*P* < 0.05), and disappears by the third year. It is also followed by a more modest increase in locality, 22.6% (*P* < 0.001), that fades quickly (Fig. 4B). But when it comes to globality, death is followed by a modest and gradual increase (a 12% rise *P* = 0.014) that continues to grow reaching a 50% increase over the baseline after 5 years (Fig. 4C).

**Figure 4:**
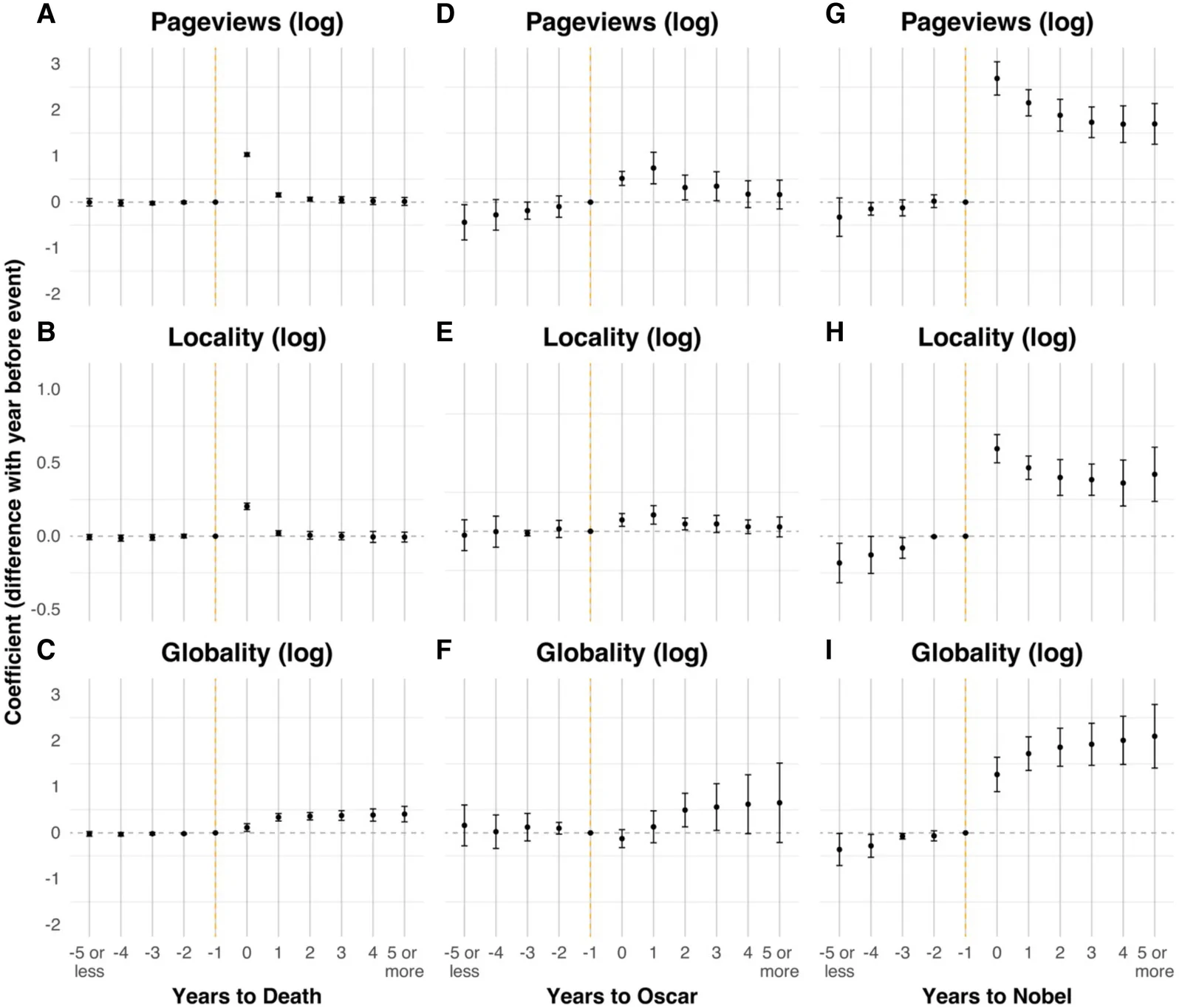
Temporal dynamics of pageviews, locality and globality in relation to three key events: death (first column), Oscar award (second column) and Nobel prize award (third column). Estimated staggered effects (two-way fixed effects) of the events on pageviews (A, D, G), locality (B, E, H), and globality (C, F, I). We show point estimates for coefficients and their 95% CI. The analysis incorporates individual and time-fixed effects, and the SE are clustered by year and individual.

Oscars follow a similar pattern with a 67.4% (*P* < 0.001) increase in pageviews in the event year (Fig. 4D) and a peak that is 109.7% (*P* < 0.001) above the baseline the year after. The effects of the award then decline, remaining positive but less pronounced for the remaining years. Oscars are also followed by a significant but more modest surge in locality (7.5% *P* = 0.0034) in the year of the event, followed by an 11% peak the year after (Fig. 4E). This effect, however, is no longer statistically significant by the fourth and fifth years. Globality again is followed by a sustained rise (Fig. 4F) that starts gradually and reaches 75 to 86% after 4 to 5 years (although with wide CI).

Finally, Nobel Prizes are followed by a more sustained effect in all three variables. In the case of pageviews, they are followed by an initial 1,369% increase (Fig. 4G), probably indicating that Nobel Prize winners are generally less famous than Oscar winners at the time of receiving an award. Unlike in the case of Oscars, the effects of a Nobel Prize remain remarkably high above the baseline even after 5 years (400%). Nobel Prizes are also followed by a large increase in locality, 81.6% (*P* < 0.001), during the award year, which wanes over time but remains significantly above pre-event levels for the entire observation period (Fig. 4H). When it comes to globality (Fig. 4I), the biographies of Nobel laureates experience a large and persistent increase in global attention, starting with a 255.6% increase in globality in the award year and reaching a 500% (*P* < 0.001) plateau by the third year.

While each event has its own particularities, they share similar qualitative patterns: spikes of attention observed for measures of pageviews and locality, and a slow and steady rise in the global attention estimated by our measure of globality.

## Discussion

In recent decades, researchers from a wide variety of fields have leveraged online biographical data to study collective memory (1, 3–6, 8–12, 16, 20, 25–27). Yet, despite significant progress, most studies do not distinguish between local and global forms of attention. Here, we contribute to this growing literature by unpacking collective memory's local and global components.

These two dimensions can help us differentiate among biographies that might look similar on the surface, like those of Johnny Unitas and Elinor Ostrom, but that hide different patterns of impact.

Unitas is an American sports legend who is remembered vividly in Louisville, Kentucky, where he played football during his college years. His impact is the story of a local hero who rose to prominence on the national stage. Ostrom's impact, by contrast, has a footprint that is less connected to her origins and more connected to her academic contributions. For instance, one of the languages contributing to Ostrom's relatively high globality is Catalan (RCA > 1 in 2015, 2016, and 2017), a language spoken in the Northeast of Spain, including many people in the city of Valencia, a fact we can connect to the idea that Ostrom wrote extensively about Valencia's Water Tribunal (*Tribunal de les Aigües de València*) (28), one of the oldest legal institutions in continuous operation in the world. The Tribunal has been running since the 10th century and represents a prime example of an institution that emerged to solve a common pool resource problem: the use of water among Valencian farmers. By separating the attention received by Unitas and Ostrom into *globality* and *locality* we honor these important differences in impact.

But while Wikipedia traffic can be insightful, it is limited by self-selection: users choose which pages to visit, often in response to current events. To address this, we designed an online trivia game where participants did not choose the biographies they were asked questions about. This approach revealed a robust local attention premium: participants were about 9 to 10% points more likely to answer questions correctly when the figure was from their own country, and 3 to 6 points more likely when they shared a language. These effects held even after accounting for global and local fame, indicating that cultural proximity exerts an independent influence on the recall of historical figures.

At the same time, the experiment allowed us to validate our online attention metrics as measures of collective memory. We found that both locality and globality scores strongly predict the probability of a correct answer, showing that these attention-based measures track the recognition behavior associated with collective memory. Figures receiving more concentrated attention in a single language (locality) or distributed attention across many languages (globality) were more likely to be correctly recognized, even by participants unfamiliar with the figure's background, further reinforcing their value as proxies for collective memory.

We also studied the impact of important events on the dynamics of collective memory, finding that these events impact locality and globality differently. These findings speak to the literature discussing how attention is affected by events such as winning prizes or death. We add to this literature by showing that globality and locality follow different dynamics. The impact of awards and death on local measures of attention is intense and short-lived, but results in a steady and more gradual increase in global attention. This suggests that these events lead to a strong communicative reverberation among local communities, which are more likely to know about the awardee or deceased before the event, and a more gradual and sustained impact among nonlocal communities.

But how might other scholars of collective memory interpret these findings? Collective memory scholars have posited that the transition from local to global memory is not merely a function of increased visibility, but a structured process shaped by commodification (29) and cultural translation (30). Commodification transforms cultural elements into marketable narratives, aligning local attention with global consumption patterns, while cultural translation adapts local narratives to global norms, making them more accessible across cultural boundaries. Our results support these ideas. The dynamics of Nobel Prize winners, for instance, seem to be celebrated locally as a triumph followed by a global canonization of a scholar's recognition in the global community. This speaks to the literature describing how commercialization and commodification help translate local narratives into global discourses (31, 32) and to the idea that certain historical figures or narratives become “glocal” (33) following major events.

Lastly, our findings highlight that Wikipedia traffic data do not merely aggregate attention but encodes systematic differences in locality and globality, revealing structured patterns of historical recall across languages. The platform functions as a digital repository of cultural memory, where attention trajectories are shaped by both user interactions and pre-existing cultural biases, consistent with evidence that shared memory is shaped by group identity (34). This suggests that historical figures who successfully navigate the tension between local significance and global resonance are more likely to sustain long-term recognition (35).

Yet, our work also has important limitations. Wikipedia attention can be used as a proxy of collective memory, but it captures behavioral traces rather than the depth of its meaning. For instance, some figures might be broadly present in collective memory but command comparatively little online attention due to a collective feeling that a figure is already well known. Also, online repositories like Wikipedia are unlikely to have strong signals about local fame in smaller languages (e.g. indigenous language with a limited population of offline speakers). These limitations are consistent with prior work showing that Wikipedia coverage is geographically uneven and shaped by cultural context, with systematic imbalances across countries, languages, and biographical subjects (36, 37).

The trivia game experiment allowed us to validate and identify our results. Yet it also suffered from some inherent limitations. The game measures recognition of basic biographical facts, not the depth, meaning, or narrative content of collective memory. A person may be widely recognized without participants knowing detailed information about them (e.g. a fan of The Beatles that does not know the date of birth of John Lennon), and different societies may remember the same figure for different reasons. Thus, the experiment validates that our attention-based measures predict recognition, and that there is a local premium associated with a shared geography and language, but it does not capture the full richness of how historical figures are interpreted, commemorated, or incorporated into a group's identity.

Our experimental dataset enjoyed wider participation in some countries (e.g. the United States, the United Kingdom, and Canada), which reflects the organic nature by which subjects arrived at our game. Also, Wikipedia language editions tend to be more comprehensively edited in some languages than others. For instance, the Spanish edition of Wikipedia has fewer articles than the Russian, French, or German editions, despite a global population of more than 600 million Spanish speakers (compared to about 250 million Russian speakers, 320 million French speakers, and 100 million German speakers). Our dataset is also limited temporally, since we could only collect information starting in 2015, limiting the temporal scope and power of our event studies. Overall, our results are limited by how data is collected, viewed, edited, and created (38–40).

Finally, our study is better at describing how local and global attention are distributed than capturing the full mechanisms through which memories remain local or become global. Processes such as education, commemoration, media circulation, translation, migration, and cultural institutions likely shape these trajectories. Our event-study results show how attention changes around major events, but they do not fully explain the social processes that produce those changes, nor their interactions (e.g. deaths can occur at times in which the news cycle is busy or more relaxed). Future work could combine large-scale attention data with surveys, curricula, media archives, or commemorative records to examine these mechanisms more directly.

In sum, we integrate online attention data from Wikipedia with experimental data from a global trivia game to disentangle the local and global components of collective memory, turning these concepts into scalable empirical measures. This decomposition revealed distinct attention-based signatures of collective memory, showing how cultural proximity, events, and fame interact with patterns of recognition and visibility. Rather than replacing qualitative, historical, or survey-based approaches, our framework complements them by revealing broad patterns of historical visibility at scale: who is remembered locally, who travels across languages, and how events reshape these trajectories over time. We hope these findings advance the study of collective memory toward a more nuanced understanding of attention, identity, and historical visibility.

## Materials and methods

### Data

We use observational data from Wikipedia and experimental data collected through an online trivia game reviewed and approved by TSE-IAST Review Board for Ethical Standards in Research, under the reference code 2022-07-001. The protocol included obtaining informed consent from each player involved in the study.

### Experimental data

We collected data by constructing an online Trivia game that we deployed on Pantheon.world, an online collective memory observatory. In the trivia game, players were asked to choose one of four options in response to a question. After playing a full game (ten questions), we asked players to voluntarily share their sociodemographic information: their sex, age range, level of education, birthplace country (for the United States, we also asked the state), and spoken languages (English, Portuguese, Spanish, Italian, French, German, Chinese, Japanese, Hindi, Russian, Polish, Mandarin, Arabic, Bengali, Indonesian, Korean, Other, Prefer not to answer). Players had 15 s to answer each question. This time limit was used to reduce the risk of players searching for the answers online. If the player did not click on any option, we registered the answer as blank.

The trivia game assigns ten biographies/questions involving historical figures from different geographies and time periods per day. Therefore, respondents do not choose the figures, their categories or their levels of globality and locality. This assignment provides exogenous exposure to biographies that may match or mismatch the respondent in terms of their geography and language. This yields an identification method for the attention premia that addresses the endogeneity concerns of web-traffic data.

From November 2022 to April 2024, we collected 414,290 answers from 41,070 players. 9,838 players shared their country of birth (24%). The data contain 20,995 blank answers and 393,295 (94.93%) nonblank answers. Questions asked basic facts (place of birth, occupation, or identifying a biography based on a short description (e.g. “John Lennon was a…” and “This musician was born in the United Kingdom in 1940…”) for the biographies of 1,581 historical figures, including 1,132 biographies of people who died before 2015. Questions for the trivia game were updated daily. For those who played the same game more than once, we kept the first answer to each question. This reduces the risk of players learning the answers by repeating the game. To ensure robustness and remove noise, we removed the questions and the historical figures that were not answered by at least five people, and we also removed the players who did not answer at least five questions. The final filtered dataset involves a sample of 3,858 unique players, 1,342 unique questions, 1,144 unique historical figures, and 41,231 responses. Our analyses link these responses to the Wikipedia-based locality and globality measures described below. This linkage is available for 40,636 responses; 15,452 of these also include participant demographics needed to construct country and language match indicators.

### Observational data

We collected the monthly and yearly pageviews of 51,465 famous historical figures corresponding to over 137.6 billion pageviews from July 2015 to July 2024 that were also available on Pantheon.world with their respective information about gender, birth year, death year (if the person is already deceased), and country of birth. The spoken language of a biography was inferred based on the official language of the individual's country of birth. Multiple languages were considered only for countries where over 25% of the population speaks more than one language, such as Hungary, where both Hungarian and English are commonly spoken.

To request the accurate number of pageviews across languages, we used the library Johnny 5 developed originally by the Collective Learning group at The MIT Media Lab (now the Center for Collective Learning) available at: (https://github.com/Datawheel/johnny5) and used to power our game. Johnny 5 checks for different names used to indicate the same historical figure across different languages. For instance, in most languages “John Lennon” is represented with the URL stub “John_Lennon,” but in Urdu is represented by “جان_لینن” and Yiddish by “דזשאן_לענאן.” Without using the adequate representation, it is not possible to accurately count pageviews across all Wikipedia language editions.

### Statistical models

#### Logit model

We use the data collected in the trivia game to assess how geographic, linguistic, and fame-related factors are associated with recognition of basic biographical facts, while controlling for confounding variables by estimating a logistic regression model described as the likelihood that a player *j* answers correctly a question about a biography *i*:


(4) $$\log\left(\;\frac{P(Correct_{ij}=1)}{1-P(Correct_{ij}\;=1)}\right)=\beta_{0\;}+\;\beta_{1}SameCountry_{\;ij}\;+\beta_{2}SameLanguage_{\;ij}\;+\beta_{3}C_{i}\;+\beta_{4}G_{i}\;+\;\beta_{5}(G_{i}\;\times \;C_{i})+\;\beta_{6\;}OlderThan40_{j}\;+\;\beta_{8}\;N\;languages_{j}+\;\beta_{7}\;Birthyear_{i}+\;\delta_{nationality_{j}}\;+\theta_{occupations_{i}}\;\;+\;\eta_{birthplace_{i}}\;\;.$$

The dependent variable $Correct_{ij}$ equals 1 when player *j* answers the question about figure *i* correctly, and 0 otherwise. $SameCountry_{ij}$ indicates whether the player *j* and figure *i* share a country of birth, and $SameLanguage_{ij}$ indicates whether the player *j* and figure *i* share at least one language in common. The language associated with the historical figure is defined by the country of birth.

Online attention prominence is captured using the locality score $C_{i}$, the globality score $G_{i}$, and their interaction $G_{i}\times C_{i}$ (combined effect of the two metrics). We include as controls for the player whether they are older than 40 ($OlderThan40_{j}$) and the number of spoken languages ($N\;languages_{j}$) and for the figure, the birth year $(Birthyear_{i}$). The terms δ,θ, and η denote fixed effects for the players' nationality and the figures' occupations and birthplace, respectively.

#### Coefficients and AMEs

The logistic model predicts the log-odds of a correct response. To interpret the magnitude of effects, the estimated coefficients can be exponentiated to calculate their contribution to the odds ratio. However, because log-odds are hard to read in substantive terms, we also report AMEs, which translate each coefficient into a change in probability.

For every observation, we compute the marginal effect implied by the fitted model—either the discrete jump in predicted probability when a binary regressor such as $SameCountry_{ij}$ switches from 0 to 1, or the slope when the regressor is continuous as for $C_{i}$ or $G_{i}$. Averaging these observation-level effects over each model's estimation sample produces the AME. In probability units, the AME for *SameCountry* is the mean difference in the probability of a correct answer between players who do and do not share the figure's country of birth; the AME for *SameLanguage* is defined similarly. For the continuous variables like $C_{i}$ or $G_{i}$, the AME gives the average percentage-point change in the likelihood of a correct answer associated with a one-unit increase in the variable.

#### Event-study model

We analyze how collective memory, used here as a proxy for collective memory, responds to major life events such as death or receiving prestigious awards (Oscars and Nobel Prizes). Using an event-study modeling framework (23, 24), we examine changes in attention surrounding these events by leveraging their staggered timing across individuals. This approach, a generalization of the difference-in-differences design, allows us to estimate the temporal dynamics of public interest and identify whether these events trigger sustained shifts in attention.

We estimate the dynamic changes in three online attention outcomes (*Y_it_*): overall Pageviews, Globality (*G_it_*), and Locality (*C_it_*) with the following equation:


(5) log(Yit)=∑k=−5k≠−15βk×Event(t=k)+αi+λt+μit

The term Event is a binary indicator set to one when the observation (*i*,*t*) occurs *k* years from the event, and zero otherwise. Formally, for each biography *i*, an event is defined as:


(6) $$\mathrm{Event}(t=k)=\{\begin{array}{cc} 1, & t-t_{i}^{\mathrm{event}}=k\\ 0, & \mathrm{otherwise} \end{array},$$

where *t* is the calendar year of observation, $t_{i}^{\mathrm{event}}$ the year in which the event occurs, and relative time from the event is $k=t-t_{i}^{\mathrm{event}}$, with *k* = 0 marking the event year. The coefficients *β_k_* estimate log-point changes relative to the baseline year, defined as the year immediately preceding the event (*k* = −1). Individual fixed effects ($\alpha_{i}$) capture time-invariant characteristics specific to each figure, while time-fixed effects ($\lambda_{t}$) account for broader temporal trends that might influence all figures in a particular year. $\mu_{it}$ is an idiosyncratic error term. For example, for a Nobel Prize awarded in 2020 (*k* = 0), the model estimates changes in attention for subsequent years (e.g. *k* = +1, +2) and preceding years (e.g. *k* = −2, −1), relative to 2019 (*k* = −1), allowing us to see if the event was followed by an increase in the three outcome variables (pageviews, globality, and locality).

In order to focus on meaningful temporal dynamics, we set a symmetric time window, spanning 5 years before and after the event. Observations beyond this range (|k|≥5) are grouped into broader categories (e.g. k≤−5 and k≥5) to preserve sample size and reduce noise. Sensitivity to this window specification is discussed in the Supplementary material.

To improve interpretability and model performance, the dependent variable is log-transformed. This reduces skewness and heteroscedasticity and allows coefficients *β_k_* to be interpreted as semielasticities. The percentage change in attention relative to the baseline year is computed as:


(7) $$\text{\%}\;\Delta \;Y=\;(\;e^{\beta_{k}}-1\;)\;\times 100\;.$$

SE are clustered at the individual and year levels to account for potential correlations within these dimensions.

## Supplementary Material

pgag308_Supplementary_Data

## Data Availability

Code and datasets are available at: https://github.com/CenterForCollectiveLearning/culturalProximityCollectiveMemory.

## References

[1] Abbas K, et al 2018. Popularity and novelty dynamics in evolving networks. Sci Rep. 8:6332. 10.1038/s41598-018-24456-2 PMC5910395 29679015

[2] Lewoniewski W, Węcel K, Abramowicz W. 2020. Modeling popularity and reliability of sources in multilingual Wikipedia. Information (Basel). 11:263.

[3] Candia C, Jara-Figueroa C, Rodriguez-Sickert C, Barabási A-L, Hidalgo CA. 2019. The universal decay of collective memory and attention. Nat Hum Behav. 3:82–91. 10.1038/s41562-018-0474-5 30932052

[4] Murray C . Human accomplishment: the pursuit of excellence in the arts and sciences, 800 B.C. to 1950; 2003. https://policycommons.net/artifacts/1299887/human-accomplishment/1903172/.

[5] Yu AZ, Ronen S, Hu K, Lu T, Hidalgo CA. 2016. Pantheon 1.0, a manually verified dataset of globally famous biographies. Sci Data. 3:150075. 10.1038/sdata.2015.75 PMC4700860 26731133

[6] García-Gavilanes R, Mollgaard A, Tsvetkova M, Yasseri T. 2017. The memory remains: understanding collective memory in the digital age. Sci Adv. 3:e1602368. 10.1126/sciadv.1602368 PMC5381953 28435881

[7] Laouenan M, et al 2022. A cross-verified database of notable people, 3500BC-2018AD. Sci Data. 9:290.

[8] Yasseri T, Gildersleve P, David L. 2022. Collective memory in the digital age. Prog Brain Res. 274:203–226. 10.1016/bs.pbr.2022.07.001 36167450

[9] Ronen S, et al 2014. Links that speak: the global language network and its association with global fame. Proc Natl Acad Sci U S A. 111:E5616–E5622. 10.1073/pnas.1410931111 PMC4284551 25512502

[10] Higham KW, Governale M, Jaffe AB, Zülicke U. 2017. Fame and obsolescence: disentangling growth and aging dynamics of patent citations. Phys Rev E. 95:042309. 10.1103/PhysRevE.95.042309 28505772

[11] Lorenz-Spreen P, Mønsted BM, Hövel P, Lehmann S. 2019. Accelerating dynamics of collective attention. Nat Commun. 10:1759. 10.1038/s41467-019-09311-w PMC6465266 30988286

[12] Jara-Figueroa C, Yu AZ, Hidalgo CA. 2019. How the medium shapes the message: printing and the rise of the arts and sciences. PLoS One. 14:e0205771. 10.1371/journal.pone.0205771 PMC6382096 30785879

[13] Schich M, et al 2014. Quantitative social science. A network framework of cultural history. Science. 345:558–562. 10.1126/science.1240064 25082701

[14] Koch P, Stojkoski V, Hidalgo CA. 2024. The role of immigrants, emigrants and locals in the historical formation of European knowledge agglomerations. Reg Stud. 58:1659–1673.

[15] Koch P, Stojkoski V, A Hidalgo C. 2024. Augmenting the availability of historical GDP per capita estimates through machine learning. Proc Natl Acad Sci U S A. 121:e2402060121. 10.1073/pnas.2402060121 PMC11441543 39284049

[16] Roediger HL, DeSoto KA. 2014. Cognitive psychology. Forgetting the presidents. Science. 346:1106–1109. 10.1126/science.1259627 25430768

[17] Candia C, Uzzi B. 2021. Quantifying the selective forgetting and integration of ideas in science and technology. Am Psychol. 76:1067–1087. 10.1037/amp0000863 34914440

[18] García-Gavilanes R, Tsvetkova M, Yasseri T. 2016. Dynamics and biases of online attention: the case of aircraft crashes. R Soc Open Sci. 3:160460. 10.1098/rsos.160460 PMC5098985 27853560

[19] West R, Leskovec J, Potts C. 2021. Postmortem memory of public figures in news and social media. Proc Natl Acad Sci U S A. 118:e2106152118. 10.1073/pnas.2106152118 PMC8463883 34526401

[20] Candia C . Quantifying the temporal dynamics of collective memory and attention in social systems. In: Handbook of computational social science. Edward Elgar Publishing Limited, 2025. p. 636–651.

[21] Hidalgo CA, Klinger B, Barabási A-L, Hausmann R. 2007. The product space conditions the development of nations. Science. 317:482–487. 10.1126/science.1144581 17656717

[22] Balassa B . 1986. Comparative advantage in manufactured goods: a reappraisal. Rev Econ Stat. 68:315–319.

[23] Miller DL . 2023. An introductory guide to event study models. J Econ Perspect. 37:203–230.

[24] Freyaldenhoven S, Hansen C, Shapiro JM. 2019. Pre-event trends in the panel event-study design. Am Econ Rev. 109:3307–3338.

[25] Fortunato S, et al 2018. Science of science. Science. 359:eaao0185.

[26] Skiena S, Ward CB. Who's bigger?: where historical figures really rank. Cambridge University Press, 2014.

[27] Candia C . Data science and the dynamics of collective memory. In: Cognition, culture, and political momentum: breaking down the silos in collective memory research. Oxford University Press, 2025. p. 270.

[28] Ostrom E . Governing the commons: the evolution of institutions for collective action. Cambridge University Press, 1990.

[29] Chen Y, Yan F, Zhang Y. 2017. Local name, global fame: the international visibility of Chinese cities in modern times. Urban Stud. 54:2652–2668.

[30] Erll A . Memory in culture. Palgrave Macmillan UK, 2011.

[31] Björkdahl A, Kappler S. 2019. The creation of transnational memory spaces: professionalization and commercialization. Int J Polit Cult Soc. 32:383–401.

[32] Olley J . 2019. Remembering Armenian music in Bolis: Komitas Vardapet in transcultural perspective. Mem Stud. 12:547–564.

[33] Strizhakova Y, Coulter R. 2019. Consumer cultural identity: local and global cultural identities and measurement implications. Int Mark Rev. 36:610–627.

[34] Coman A, Hirst W. 2015. Social identity and socially shared retrieval-induced forgetting: the effects of group membership. J Exp Psychol Gen. 144:717–722. 10.1037/xge0000077 25938179

[35] Foroughi H . 2020. Collective memories as a vehicle of fantasy and identification: founding stories retold. Organ Stud. 41:1347–1367.

[36] Tripodi F . 2023. Ms. Categorized: gender, notability, and inequality on Wikipedia. New Media Soc. 25:1687–1707.

[37] Callahan ES, Herring SC. 2011. Cultural bias in Wikipedia content on famous persons. J Am Soc Inf Sci Technol. 62:1899–1915.

[38] Brand D . 2020. Algorithmic decision-making and the law. JeDEM—eJournal EDemocracy Open Gov. 12:114–131.

[39] Yarger L, Cobb Payton F, Neupane B. 2019. Algorithmic equity in the hiring of underrepresented IT job candidates. Online Inf Rev. 44:383–395.

[40] Lamprecht D, Lerman K, Helic D, Strohmaier M. 2017. How the structure of Wikipedia articles influences user navigation. New Rev Hypermedia Multimed. 23:29–50. 10.1080/13614568.2016.1179798 PMC5468769 28670171

