# Supplementary material for: Cultural proximity reveals a local premium in collective memory
Source: PNAS Nexus. 2026 Sep 21;5(10):pgag308. doi: 10.1093/pnasnexus/pgag308 (PMC13641167; doi:10.1093/pnasnexus/pgag308)
Supplement: Supplementary file 1 [file pgag308_supplementary_data.pdf]

Supplementary Material:

Cultural Proximity Reveals a Local Premium in Collective Memory

Mariana Macedo[4], Melanie Oyarzun[3], Cristian Candia[5,6], César A. Hidalgo[1,2,3,*]

[1] Center for Collective Learning, IAST, Toulouse School of Economics, Université de Toulouse Capitole, Toulouse, France
[2] Alliance Manchester Business School, University of Manchester, Manchester, United Kingdom
[3] Center for Collective Learning, CIAS, Corvinus University of Budapest, Budapest, Hungary
[4] School of Computer Science and Engineering, Constructor University, Bremen, Germany
[5] Instituto de Data Science, Facultad de Ingeniería, Universidad del Desarrollo, Las Condes, 7610658, Chile.
[6] Northwestern Institute on Complex Systems (NICO), Northwestern University, Evanston, IL 60208, USA

*to whom correspondence should be addressed: cesifoti@gmail.com

This supplementary material provides additional data and methodological information related to our main manuscript.

Observational data collection

We collected data about 51,465 famous popular biographies on Wikipedia from July 2015 to July 2024. Our data utilized information, data, and methods available on Pantheon.world and by Laouenan et al.[1] complementing our dataset with sociodemographic data (e.g. gender and age). We used a Python library called johnny5 to match biography page names across different languages, ensuring that we accurately identify the corresponding pages for the same individual.

To ensure data quality, we excluded profiles that had any month with zero pageviews between 2020 and 2022, as well as those with fewer than 1,000 total pageviews over all the years. This approach ensures that we retain only profiles with consistent attention over the analyzed period, eliminate potential gaps caused by internet outages or data collection issues, and exclude recently created profiles that may not have had sufficient time to accumulate pageviews. The resulting dataset consists of 51,465 biographies (out of the 158,170 biographies in Pantheon) including 27,705 biographies of individuals who were still alive in 2024. Tables SM1 and SM2 tabulate the

[1] Laouenan et al., "A Cross-Verified Database of Notable People, 3500BC-2018AD."

number of historical figures available on the top 10 most common birth countries (Table SM1) and occupations (Table SM2).

When examining local and global fame, Figure SM1 highlights the distribution of highly globally famous biographies in our dataset. A significant proportion of these globally renowned figures originate from the United States and central European countries, whereas countries in Africa and parts of South America, such as those in the Western region, are underrepresented. This stark contrast underscores the geographic disparities in global fame.

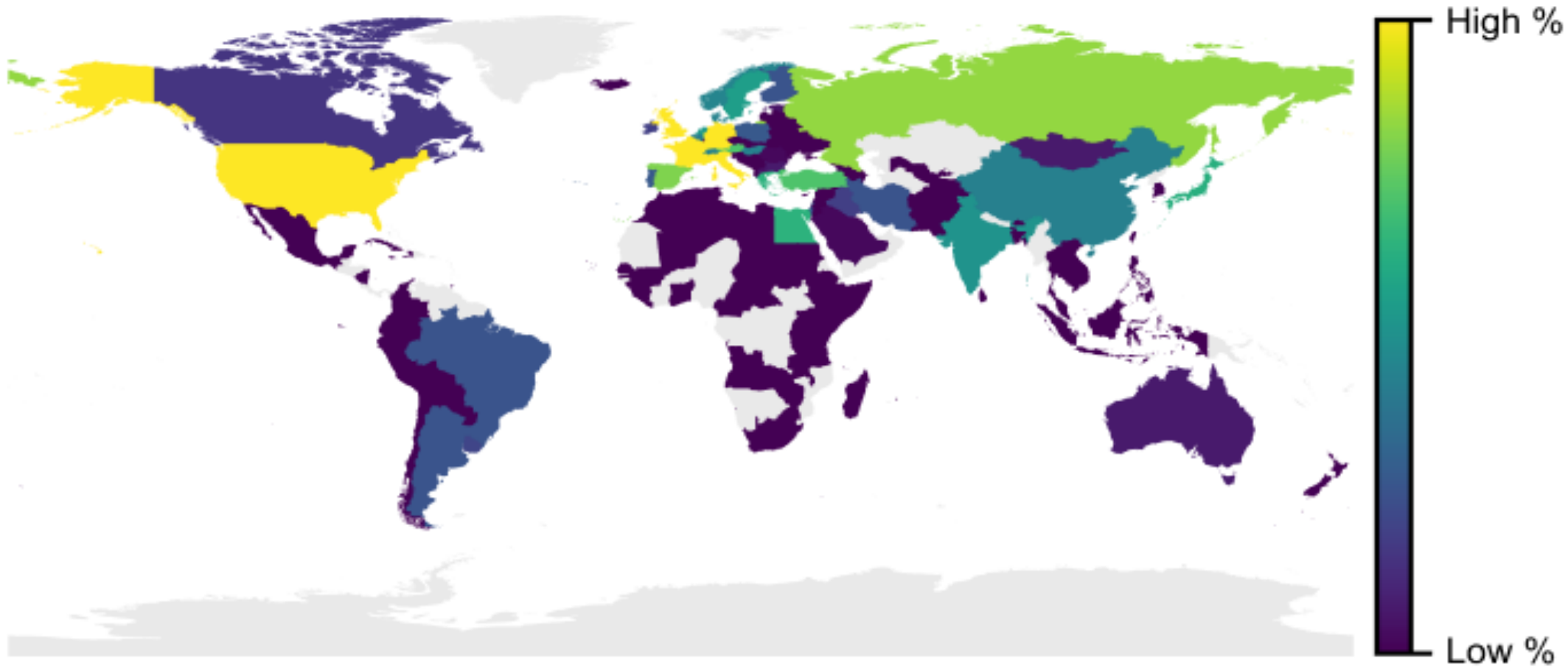

Figure SM1: Geographical distribution of the highly globally famous biographies in our sample. The United States, Europe, and Russia show a higher concentration of biographies compared to other regions, reflecting historical patterns in the development of collective memory and fame.

Table SM1: Percentage (number of people) that are from each birth country in our dataset. The top 10 birth countries are in relation to the already dead historical figures.

Birth country	All	Dead	Alive
United States	21.70% (10774)	18.46% (3616)	23.35% (6291)
Germany	8.36% (4151)	12.82% (2511)	5.56% (1499)
France	6.15% (3055)	9.77% (1914)	3.75% (1011)
United Kingdom	8.11% (4030)	9.02% (1767)	7.29% (1965)
Italy	4.71% (2342)	6.82% (1336)	3.20% (863)
Russia	2.23% (1110)	2.84% (557)	1.94% (522)
Spain	3.04% (1511)	2.84% (557)	3.27% (881)
Japan	6.43% (3196)	2.05% (402)	10.11% (2723)
Austria	1.10% (550)	1.72% (338)	0.72% (195)
China	1.31% (654)	1.60% (314)	1.06% (285)

Table SM2: Percentage (number of people) that are from each occupation in our dataset. The top 10 occupations are in relation to the already dead historical figures.

Occupations	All	Dead	Alive
Politician	14.57% (8258)	23.72% (5616)	6.66% (1955)
Writer	6.95% (3938)	11.38% (2695)	3.18% (933)
Actor	11.33% (6420)	6.28% (1489)	15.23% (4469)
Religious Figure	2.32% (1312)	4.03% (954)	0.69% (202)
Soccer Player	17.70% (10031)	3.96% (937)	29.91% (8775)
Military Personnel	1.74% (988)	3.73% (883)	0.16% (47)
Painter	1.58% (894)	3.49% (826)	0.17% (50)
Composer	1.59% (902)	2.91% (688)	0.57% (166)
Musician	3.11% (1765)	2.22% (526)	3.67% (1078)
Philosopher	1.09% (621)	2.21% (523)	0.20% (59)

1.1 Distribution of occupations and countries

We observe in Tables SM1-SM2 that certain occupations and countries are more prominently represented in our Wikipedia sample, serving as a proxy for how professions have evolved across countries over time. Here, we illustrate how the measures of globality and locality vary across occupations and countries when considering individuals who died before 2015 and those still alive at that time (Figure SM2). For instance, John Lennon is more locally than globally famous; Claude Monet is more globally recognized than Ada Lovelace but less locally prominent; and William Shakespeare stands out as both highly global and local in fame. Among living figures, Joe Biden, Emmanuel Macron, and Donald Trump are all globally recognized, with Trump showing significantly higher local recognition than Macron or Biden. These examples suggest that certain occupations (e.g. Politician) and countries (e.g. United States) are more likely to have a higher proportion of biographies that are either highly local or highly global (Figure SM3-SM4). They also reveal a clustering effect, where individuals born in similar periods tend to share similar patterns of fame. Therefore, when analyzing occupations and countries, we observe generational shifts in recognition.

Italy and France show greater historical prominence, particularly among deceased individuals, while countries like Japan are gaining increased recognition among those still alive (Figure SM5). Table SM3 shows that the average birth year of famous biographies from Italy is 1839 and from France 1862, whereas for Japan it is much more recent, 1966. Interesting patterns emerge when comparing highly global and less global biographies. The average birth year for highly global biographies is earlier in Italy and France, and later for countries like Brazil and Japan, highlighting generational differences in the emergence of global fame.

Table SM3: Average birth year (with standard deviation) for all famous biographies as well as for those classified as highly global and less global.

**Birth Country**	**Average Year**		
	**All Biographies**	**High Global Biographies**	**Low Global Biographies**
Brazil	1958 (±35.53)	1963 (±28.92)	1951 (±35.56)
France	1862 (±133.57)	1821 (±129.83)	1940 (±65.89)
Germany	1875 (±118.42)	1850 (±123.16)	1890 (±102.08)
Italy	1839 (±182.76)	1769 (±201.12)	1926 (±91.25)
Japan	1966 (±62.25)	1961 (±48.63)	1937 (±104.73)
United Kingdom	1905 (±107.38)	1873 (±120.38)	1929 (±90.53)
United States	1940 (±42.91)	1925 (±43.65)	1945 (±40.72)

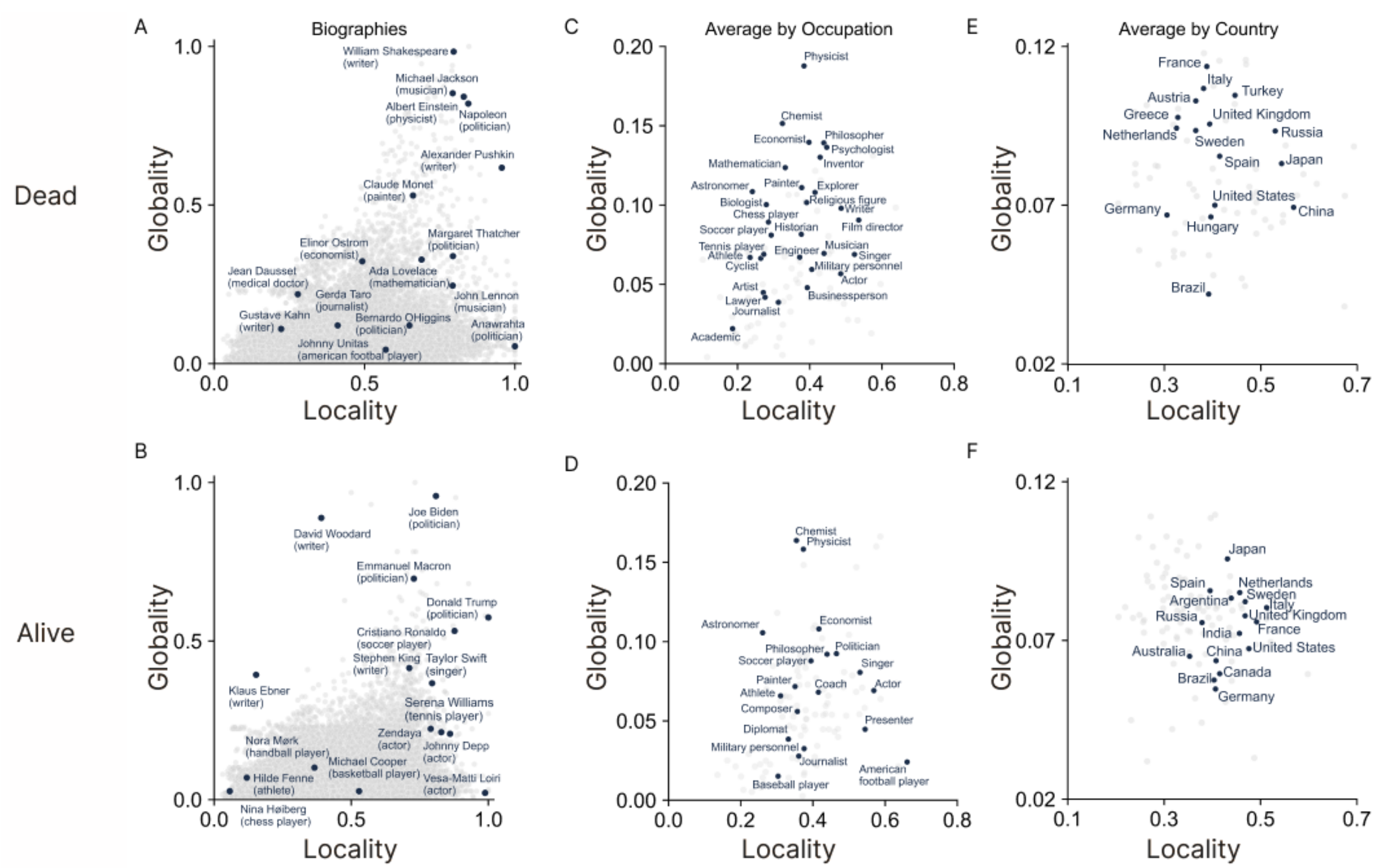

Figure SM2: Replication of Figure 2 from the manuscript to examine local and global collective memory, based on our dataset of historical figures who died before 2015. The first row presents data for deceased individuals, and the second row for those still alive. *(A–B) Scatter plots of locality and globality with selected examples: (A) biographies of individuals who died before 2015, and (B) those still alive as of 2015. (C–D) Average locality and globality by occupation: (C) for deceased individuals and (D) for living individuals. (E–F) Average locality and globality by country of birth: (E) for deceased individuals and (F) for living individuals.*

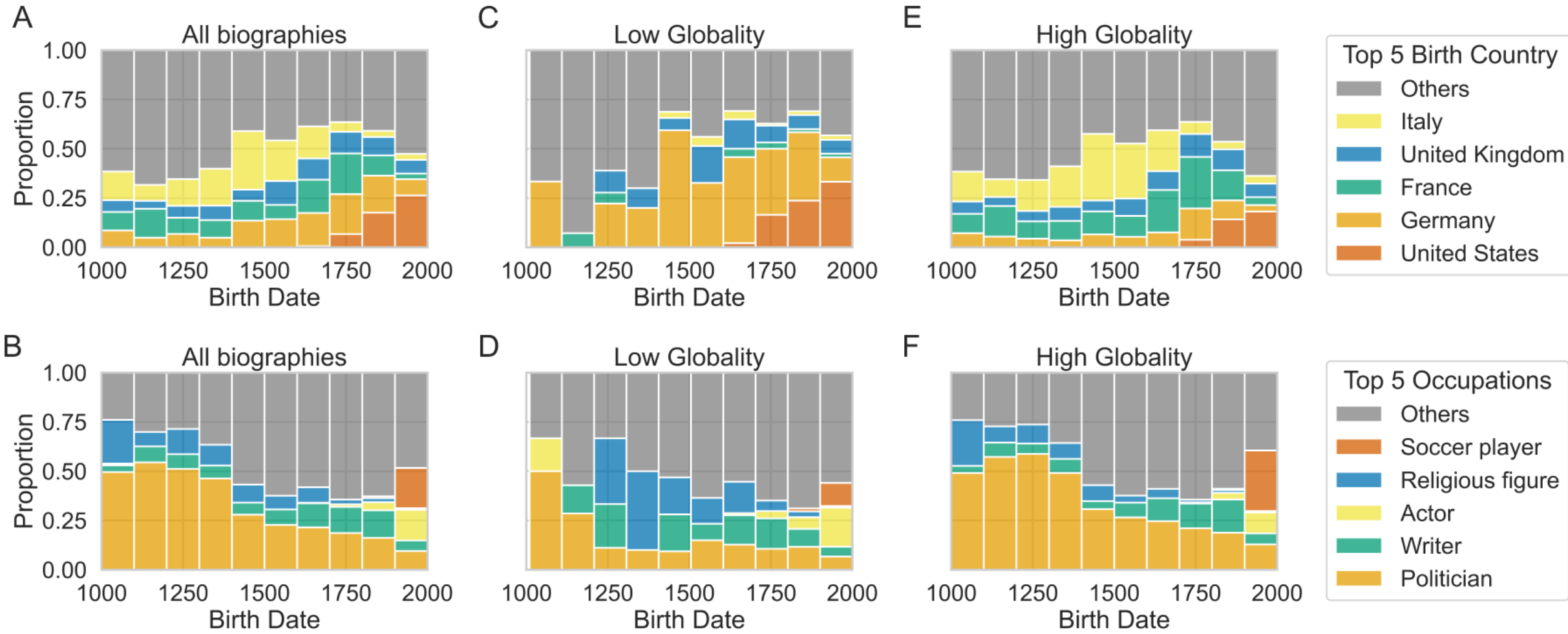

Figure SM3: Proportion of biographies by birth year for the top five countries of birth and occupations, categorized by levels of globality.

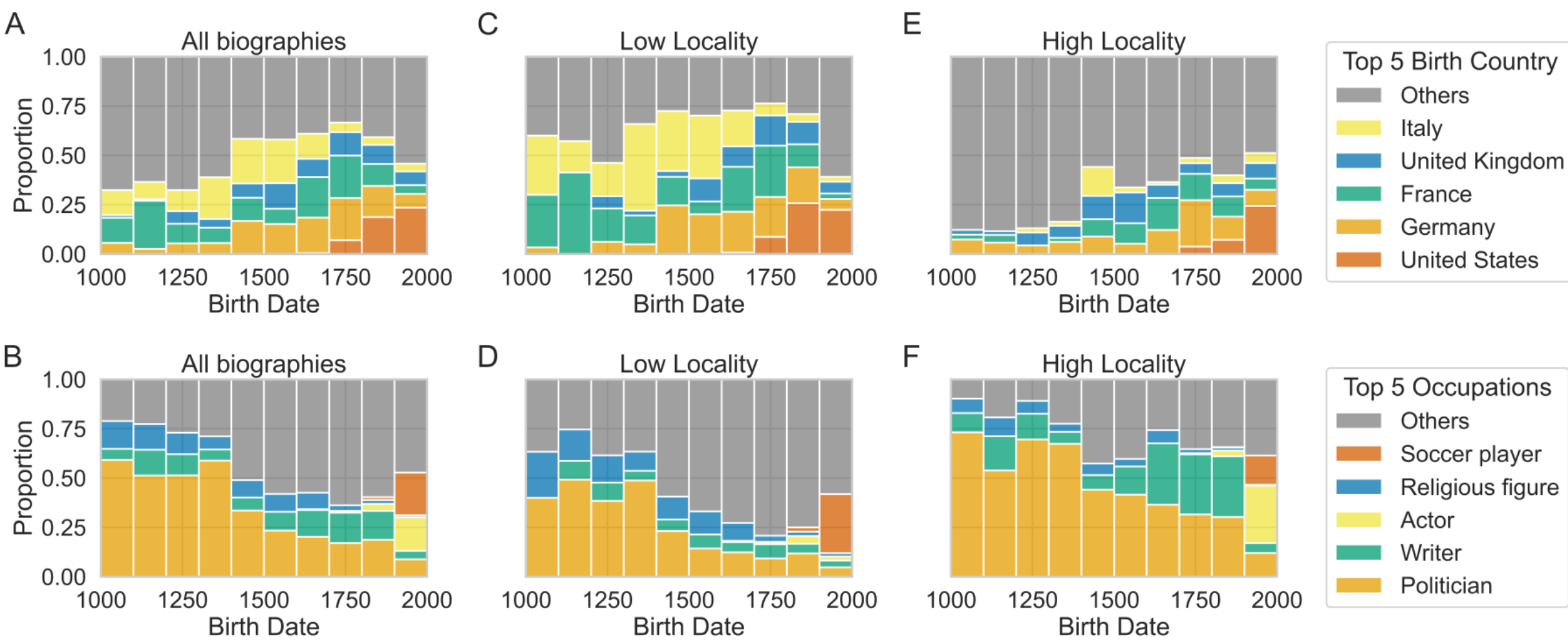

Figure SM4: Proportion of biographies by birth year for the top five countries of birth and occupations, categorized by levels of locality.

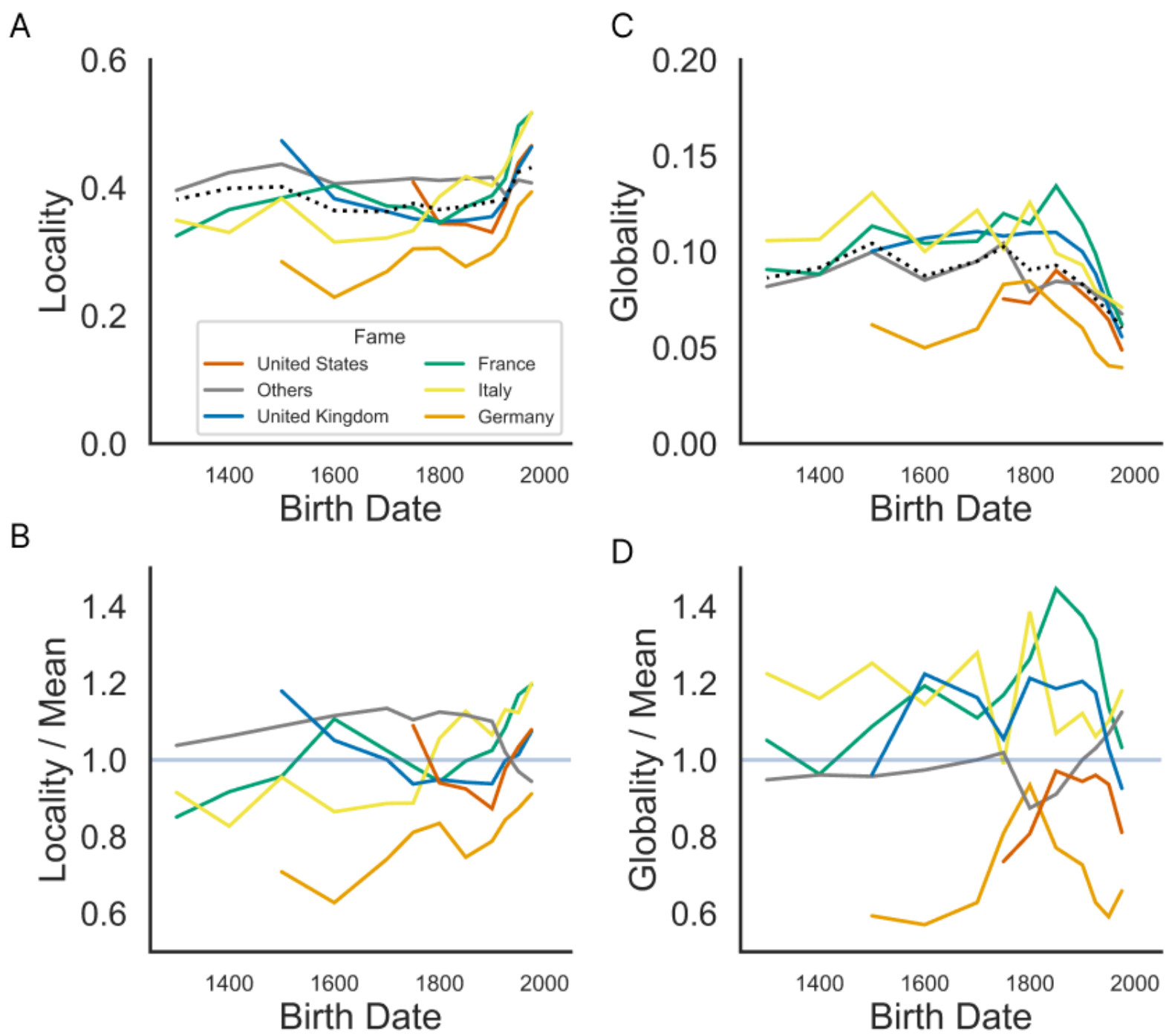

Figure SM5: Average locality and globality measures over time aggregated for the top five countries. *A black dotted line in panels (A) and (C) represents the overall global average across all countries. A horizontal line at y = 1 in panels (B) and (D) marks the global benchmark—values above this line indicate higher-than-expected locality or globality. (A) and (C) show the average locality and globality values for the top five countries, with all other countries grouped together. (B) and (D) present normalised values of locality and globality, calculated relative to the overall global averages.*

Models of the distribution of occupations and countries

We examine how the distribution of individuals across occupations and countries relates to both locality and globality. First, we focus on occupations within each biographies' birth country. For year t, let $\bar{Y}_{bt}$ be the country-level average of either the Globality or Locality metric. We explain $\bar{Y}_{bt}$ as a function of the fraction of individuals in each major occupation f_{obt}—particularly those within the top 10—in that country:

$$\bar{Y}_{bt} = \beta_0 + \beta_1 f_{Politician,bt} + \beta_2 f_{Writer,bt} + \beta_2 f_{Actor,bt} + \beta_3 f_{Religious,bt} + \beta_4 f_{Soccer,bt} + \beta_5 f_{Painter,bt} + \beta_6 f_{Composers,bt} + \beta_7 f_{Musicians,bt} + \beta_8 f_{Philosophers,bt} + \beta_9 f_{military,bt} + \cdots + \beta_k f_{occupations,bt} + \lambda_t + \epsilon_{bt} \quad (1)$$

where λ_t is a year fixed effect and ϵ_{bt} is the error term.

Next, we look at how the fraction of individuals from each country influences the average global or local fame of a specific occupation. Denote $\bar{Y}_{ot}$ the occupation-level average (**across countries**) of globality and locality for occupation *o* in year *t*. We then have:

$$\bar{Y}_{ot} = \alpha_0 + \alpha_1 f_{US,ot} + \alpha_2 f_{DE,ot} + \alpha_2 f_{FR,ot} + \alpha_3 f_{UK,ot} + \alpha_4 f_{IT,ot} + \alpha_5 f_{RU,ot} + \alpha_6 f_{sp,ot} + \alpha_7 f_{JP,ot} + \alpha_8 f_{AU,ot} + \alpha_9 f_{CH,ot} + \cdots + \alpha_k f_{countries,ot} + \lambda_t + \epsilon_{ct} \quad (2)$$

Our results shown in Tables SM4-SM7 reveal that the distribution of occupations **within** countries is more influential than the distribution of occupations **across** countries. Specifically, for the within-country models, we explain 28.5% of the variance in globality and 25.9% in locality whereas focusing on countries alone explains only 17.4% (global) and 18.0% (local). Notably, only a subset of occupations significantly predicts globality. When we restrict the analysis to the top 10 countries, the variance explained by within-country distributions increases to 42.9% for global and 44.5% for local. Similarly, focusing on the top 10 occupations, the variance explained by across-country distributions goes to 34% for global and 36.2% for local. Together, these findings indicate that the distribution of historical figures across occupations and countries plays a role for the local and global dimensions.

Table SM4: OLS model for global fame of countries using the United States as reference.

	(All data)	(Top 10 Occupations)	(Top 10 Countries)	(Top 10 Both)
Germany	-0.008^{***}	0.017^{***}	-0.005^{***}	0.005^{**}
	(0.002)	(0.004)	(0.002)	(0.003)
United Kingdom	0.005^{***}	0.026^{***}	0.008^{***}	0.013^{***}
	(0.002)	(0.004)	(0.002)	(0.003)
Japan	-0.011^{***}	0.016^{***}	-0.008^{***}	-0.001
	(0.002)	(0.004)	(0.002)	(0.003)
France	0.013^{***}	0.047^{***}	0.016^{***}	0.033^{***}
	(0.002)	(0.004)	(0.002)	(0.003)
Italy	0.011^{***}	0.046^{***}	0.015^{***}	0.032^{***}
	(0.002)	(0.004)	(0.002)	(0.003)
Brazil	-0.030^{***}	-0.000	-0.027^{***}	-0.020^{***}
	(0.002)	(0.004)	(0.002)	(0.003)
Spain	0.003	0.040^{***}	0.007^{***}	0.021^{***}
	(0.002)	(0.004)	(0.002)	(0.003)
Russia	0.013^{***}	0.039^{***}	0.016^{***}	0.020^{***}
	(0.002)	(0.004)	(0.002)	(0.003)
India	-0.013^{***}	0.022^{***}	-0.009^{***}	0.002
	(0.002)	(0.004)	(0.002)	(0.003)
Canada	-0.011^{***}	0.013^{***}	-0.007^{***}	-0.007^{***}
	(0.002)	(0.004)	(0.002)	(0.003)
Intercept	0.050^{***}	0.019^{***}	0.046^{***}	0.042^{***}
	(0.001)	(0.004)	(0.002)	(0.003)
Control by Year	Yes	Yes	Yes	Yes
Other occupations	Yes	No	Yes	No
Other countries	Yes	Yes	No	No
Observations	32,883	10,778	8,566	1,210
R^2	0.174	0.340	0.127	0.426
Adjusted R^2	0.168	0.326	0.125	0.416
Residual Std. Error	0.035 (df=32647)	0.028 (df=10544)	0.035 (df=8545)	0.020 (df=1189)
F Statistic	29.259^{***} (df=235; 32647)	23.354^{***} (df=233; 10544)	62.081^{***} (df=20; 8545)	44.060^{***} (df=20; 1189)
Observations	32,883	10,778	8,566	1,210

*p<0.1; **p<0.05; ***p<0.01

Table SM5: OLS model for local fame of countries using the United States as reference.

	(All data)	(Top 10 Occupations)	(Top 10 Countries)	(Top 10 Both)
Germany	-0.052***	-0.026	-0.052***	-0.022*
	(0.007)	(0.019)	(0.006)	(0.013)
United Kingdom	-0.016**	0.007	-0.016***	0.011
	(0.007)	(0.019)	(0.006)	(0.013)
Japan	0.151***	0.153***	0.151***	0.159***
	(0.008)	(0.020)	(0.007)	(0.013)
France	0.020***	0.041**	0.020***	0.045***
	(0.007)	(0.019)	(0.007)	(0.013)
Italy	0.032***	0.038**	0.031***	0.043***
	(0.008)	(0.019)	(0.007)	(0.013)
Brazil	0.009	0.012	0.008	0.018
	(0.008)	(0.021)	(0.007)	(0.013)
Spain	0.032***	0.025	0.031***	0.031**
	(0.008)	(0.020)	(0.008)	(0.013)
Russia	0.071***	0.109***	0.070***	0.115***
	(0.008)	(0.020)	(0.007)	(0.013)
India	-0.009	0.027	-0.010	0.033**
	(0.008)	(0.021)	(0.008)	(0.013)
Canada	-0.044***	-0.068***	-0.045***	-0.062***
	(0.008)	(0.021)	(0.007)	(0.013)
Intercept	0.388***	0.413***	0.397***	0.421***
	(0.006)	(0.018)	(0.007)	(0.013)
Year	Yes	Yes	Yes	Yes
Other occupations	Yes	No	Yes	No
Other countries	Yes	Yes	No	No
Observations	32,883	10,778	8,566	1,210
R^2	0.180	0.362	0.143	0.276
Adjusted R^2	0.174	0.347	0.141	0.264
Residual Std. Error	0.149 (df=32647)	0.132 (df=10544)	0.133 (df=8545)	0.097 (df=1189)
F Statistic	30.415*** (df=235; 32647)	25.633*** (df=233; 10544)	71.565*** (df=20; 8545)	22.682*** (df=20; 1189)
Observations	32,883	10,778	8,566	1,210

*p<0.1; **p<0.05; ***p<0.01

Table SM6: OLS model for global fame of occupations using Soccer Players as reference.

	(All data)	(Top 10 Occupations)	(Top 10 Countries)	(Top 10 Both)
Politician	0.021***	0.021***	0.020***	0.013***
	(0.001)	(0.001)	(0.004)	(0.003)
Actor	-0.007***	-0.006***	0.001	-0.006*
	(0.001)	(0.001)	(0.004)	(0.003)
Writer	0.012***	0.013***	0.031***	0.020***
	(0.001)	(0.001)	(0.004)	(0.003)
Athlete	-0.004***	-0.003***	0.009**	-0.005
	(0.001)	(0.001)	(0.004)	(0.003)
Singer	0.018***	0.018***	0.021***	0.007**
	(0.001)	(0.001)	(0.004)	(0.003)
Musician	0.007***	0.008***	0.025***	0.011***
	(0.002)	(0.002)	(0.004)	(0.003)
Religious Figure	0.015***	0.016***	0.025***	0.011***
	(0.001)	(0.001)	(0.004)	(0.003)
Military Personnel	-0.002	-0.001	0.010**	-0.005
	(0.002)	(0.002)	(0.004)	(0.003)
Composer	0.001	0.002	0.040***	0.025***
	(0.002)	(0.002)	(0.004)	(0.003)
Painter	0.007***	0.008***	0.043***	0.028***
	(0.002)	(0.002)	(0.004)	(0.003)
Intercept	0.043***	0.040***	0.025***	0.040***
	(0.001)	(0.001)	(0.003)	(0.003)
Year	Yes	Yes	Yes	Yes
Other occupations	Yes	No	Yes	No
Other countries	Yes	Yes	No	No
Observations	32,883	10,778	8,566	1,210
R^2	0.285	0.131	0.459	0.249
Adjusted R^2	0.282	0.129	0.451	0.236
Residual Std. Error	0.032 (df=32753)	0.031 (df=10757)	0.027 (df=8438)	0.022 (df=1189)
F Statistic	101.257*** (df=129; 32753)	80.755*** (df=20; 10757)	56.386*** (df=127; 8438)	19.686*** (df=20; 1189)
Observations	32,883	10,778	8,566	1,210

*p<0.1; **p<0.05; ***p<0.01

Table SM7: OLS model for local fame of occupations using Soccer Players as reference.

	(All data)	(Top 10 OCC)	(Top 10 CON)	(Top 10 Both)
Politician	0.074***	0.075***	0.048***	0.054***
	(0.005)	(0.005)	(0.015)	(0.011)
Actor	0.171***	0.169***	0.184***	0.190***
	(0.006)	(0.006)	(0.015)	(0.011)
Writer	0.116***	0.114***	0.061***	0.071***
	(0.006)	(0.006)	(0.015)	(0.011)
Athlete	-0.095***	-0.097***	-0.086***	-0.074***
	(0.006)	(0.006)	(0.016)	(0.011)
Singer	0.149***	0.148***	0.159***	0.172***
	(0.006)	(0.006)	(0.016)	(0.011)
Musician	0.082***	0.079***	0.079***	0.092***
	(0.007)	(0.007)	(0.016)	(0.011)
Religious Figure	-0.003	-0.006	-0.026	-0.013
	(0.006)	(0.006)	(0.016)	(0.012)
Military Personnel	0.026***	0.023***	-0.014	-0.001
	(0.007)	(0.007)	(0.016)	(0.012)
Composer	-0.026***	-0.028***	-0.014	-0.001
	(0.007)	(0.007)	(0.016)	(0.012)
Painter	0.023***	0.020***	0.021	0.035***
	(0.008)	(0.008)	(0.016)	(0.012)
Intercept	0.383***	0.385***	0.420***	0.410***
	(0.005)	(0.006)	(0.013)	(0.011)
Year	Yes	Yes	Yes	Yes
Other occupations	Yes	No	Yes	No
Other countries	Yes	Yes	No	No
Observations	32,883	10,778	8,566	1,210
R^2	0.259	0.251	0.445	0.465
Adjusted R^2	0.256	0.249	0.437	0.456
Residual Std. Error	0.142 (df=32753)	0.141 (df=10757)	0.108 (df=8438)	0.083 (df=1189)
F Statistic	88.723*** (df=129; 32753)	180.055*** (df=20; 10757)	53.365*** (df=127; 8438)	51.736*** (df=20; 1189)
Observations	32,883	10,778	8,566	1,210

*p<0.1; **p<0.05; ***p<0.01

Experimental data

We collected data through an online trivia game hosted on Pantheon.world, a digital observatory of collective memory. Players answered multiple-choice questions about historical figures, selecting one of four possible answers. To dissuade players from searching for the answers online, each question was time-limited to 15 seconds, with unanswered questions recorded as blank responses. After completing the game, participants were invited to voluntarily provide sociodemographic information, including sex (female, male, other, prefer not to answer), age range (in bins of ten (e.g. 20-29, 30-39, etc.)), education level, country of birth (and U.S. state, if applicable), and spoken languages (English, Portuguese, Spanish, Italian, French, German, Chinese, Japanese, Hindi, Russian, Polish, Mandarin, Arabic, Bengali, Indonesian, Korean, Other, or Prefer not to answer).

Using this dataset, we examined the likelihood of participants answering correctly questions about biographies when the birthplace of the biography matched the self-reported place of origin of the player (same country, or *local*) or when these two attributes did not match (different country, or *foreign*). In the manuscript, we present the overall results, and in Figure SM6, we extend this analysis by comparing the biographies of figures who passed away before 2015 with those who are still alive. Our findings suggest that for living historical figures, global recognition plays a more prominent role than for deceased figures. Additionally, the accuracy gap between locals and foreigners is smaller for highly globally recognized individuals, indicating that modern fame, likely amplified by digital connectivity, may transcend national boundaries more than in the past.

In Figure SM7, we compare the likelihood that players correctly answer questions about historical figures who do—or do not—share their spoken language. The results mirror earlier patterns observed with shared nationality, showing a marked increase in accuracy for figures with higher levels of both globality and locality. These findings suggest that cultural proximity—whether reflected through language or nationality—plays a significant role in the recognition of historical figures.

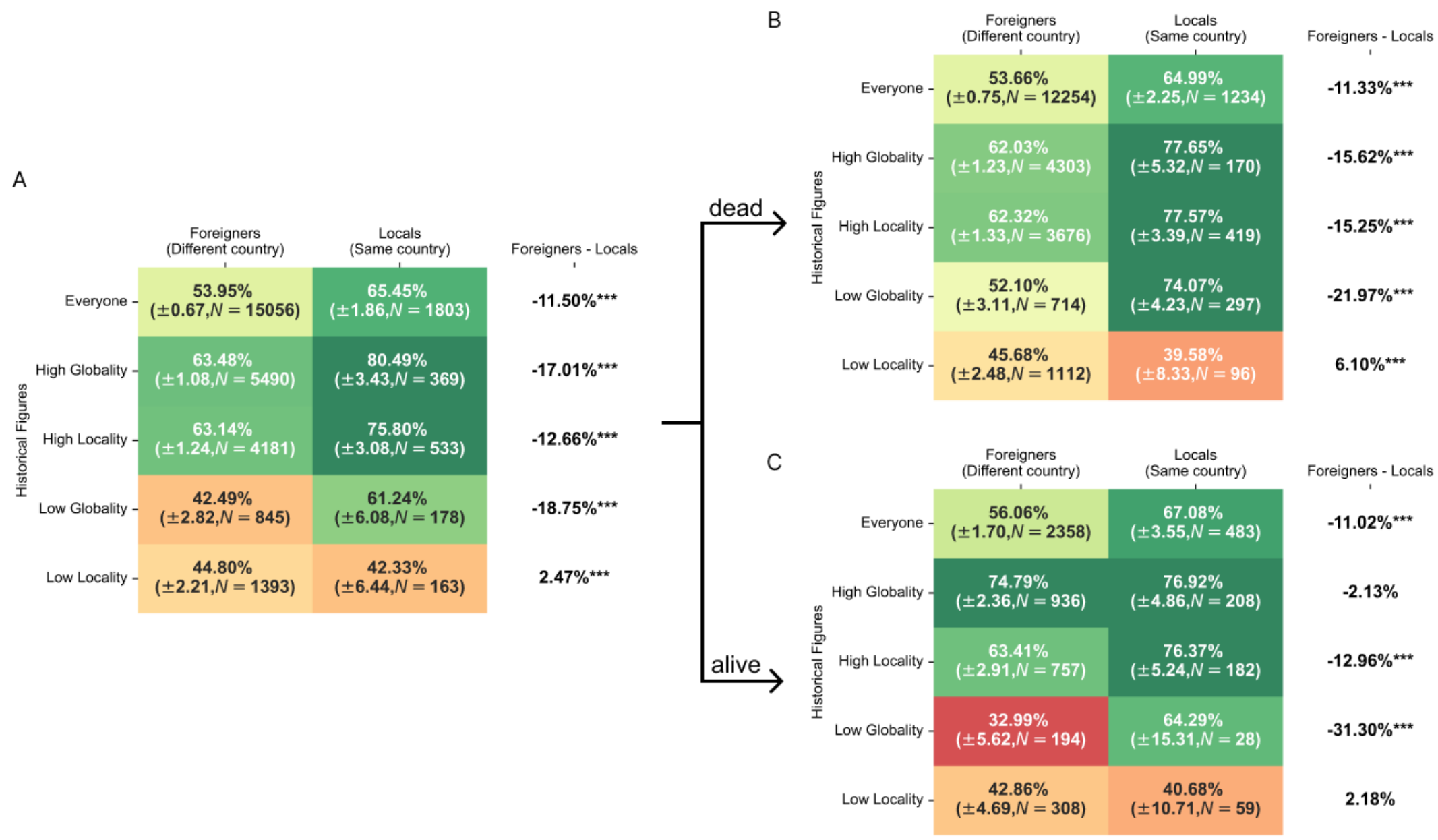

Figure SM6: Replication of Figure 3 of the manuscript for all our sample (A, all) considering only our dataset of historical figures that died before 2015 (B, dead) and figures that are still alive (C, alive). Living individuals show stronger global recognition effects than deceased figures.

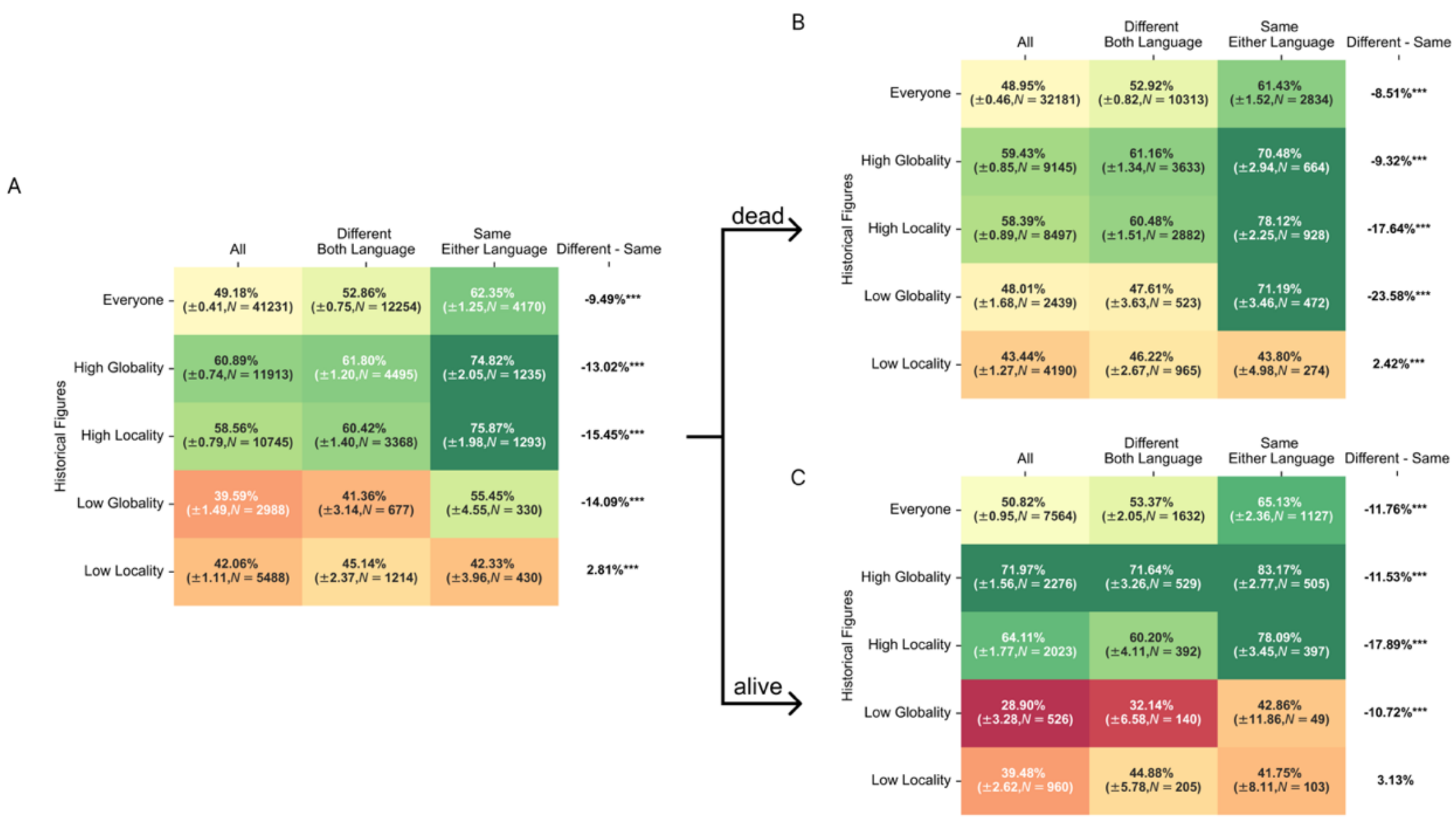

Figure SM7: Replication of Figure SM6 considering the shared official language instead of the nationality. Cultural proximity and levels of globality and locality influence the likelihood of individuals recognizing information about popular biographies.

We then estimate the likelihood that a player j recognizes a historical figure i using a logistic regression reported as Equation 3.

$$\log\left(\frac{P(Correct_{ij}=1)}{1-P(Correct_{ij}=1)}\right) = \beta_0 + \beta_1 SameCountry_{ij} + \beta_2 SameLanguage_{ij} + \beta_3 C_i + \beta_4 G_i + \beta_5 (G_i \times C_i) + \beta_6\, OlderThan40_j + \beta_8\, N\ languages_j + \beta_7\, Birthyear_i + \delta_{nationality_j} + \theta_{occupations_i} + \eta_{birthplace_i} \quad (3)$$

Here, the term $SameCountry_{ij}$ is equal to 1 when the player's stated nationality coincides with the figure's country of birth, and $SameLanguage_{ij}$ which equals 1 when they share at least one language (player-reported languages matched to those spoken in the figure's birth country). We include the figure locality score C_i, the globality score G_i and their interaction $G_i\ x\ C_i$. We also add control variables to isolate the incremental role of these variables: for the player we consider whether they are older than 40 ($OlderThan40_j$) and the number of spoken languages ($N\ languages_j$) and for the figure, the birth year ($Birthyear_i$). On the historical figure side, we add birth-year (linear), a full set of occupation dummies, and birthplace fixed effects; on the player side, we account for being older than forty, the self-reported number of spoken languages, and nationality dummies. The terms δ, θ and η denote fixed effects for the players' nationality and the figures' occupations and birthplace respectively.

The main Equation 3 results are shown in Table 1 of the manuscript, and we present here four additional robustness checks: Table SM8 re-estimates the baseline on the full sample and on the subset with complete demographics; Table SM9 adds gender and the exact number of languages spoken; Table SM10 analyses the alternative interaction terms between proximity and collective-memory scores; and Table SM11 presents predicted probabilities derived from the preferred model for easier interpretation, as visualized in Figure SM8. The sub-sample without demographic data is also included for reference. Across all model specifications, sharing a country or language with the figure, as well as the effects of the local and global collective memory metrics, remain consistently positive and statistically significant, reinforcing the robustness of our main conclusions.

Table SM8*. *Logit model estimations of the likelihood that participants correctly answer a question about a historical figure, comparing full sample and subsample with only demographic information. *Each column reports a different specification, including a subset of the explanatory variables.* ***Panel A*** *displays the logit coefficients (indicating changes in log-odds) and standard errors in parentheses.* ***Panel B*** *reports the estimated average marginal effects (AMEs) for the main variables, computed by averaging observation-level marginal effects over each model's estimation sample. For globality and locality, we indicate the average change in the probability of a correct response per one-unit increase in these scores (dY/dX). Significance levels: * p < 0.05, ** p < 0.01, *** p < 0.001.*

	Full sample					With Demographic information				
	**(1)**	**(2)**	**(3)**	**(4)**	**(5)**	**(6)**	**(7)**	**(8)**	**(9)**	**(10)**
**A: (LOG) ODDS RATIO OF CORRECT ANSWER**										
Locality	1.806***		0.582***	0.673***	-0.006	2.342***		1.074***	1.174***	0.183
	(0.075)		(0.138)	(0.147)	(0.176)	(0.121)		(0.224)	(0.238)	(0.288)
Globality		2.482***	0.006	0.239	0.181		2.437***	-0.084	0.130	0.526
		(0.068)	(0.379)	(0.392)	(0.463)		(0.105)	(0.577)	(0.594)	(0.714)
Locality X Globality			3.623***	3.246***	4.092***			3.589***	3.225***	3.657***
			(0.599)	(0.620)	(0.708)			(0.911)	(0.938)	(1.086)
Birth year				-0.000	0.000**				-0.000	0.000
				(0.000)	(0.000)				(0.000)	(0.000)
**B: AVERAGE MARGINAL EFFECTS**										
Locality	0.445***		0.342***	0.343***	0.222***	0.566***		0.463***	0.466***	0.250***
	(0.018)		(0.019)	(0.020)	(0.031)	(0.028)		(0.030)	(0.031)	(0.048)
Globality		0.599***	0.488***	0.494***	0.581***		0.582***	0.459***	0.462***	0.597***
		(0.015)	(0.019)	(0.019)	(0.026)		(0.024)	(0.028)	(0.028)	(0.039)
Num.Obs.	40884	40884	40884	40636	40636	16736	16736	16736	16652	16652
AIC	56074.1	55191.4	54880.4	54566.3	53807.6	22641.8	22414.2	22187.7	22090.7	21785.5
BIC	56091.3	55208.7	54914.8	54609.4	54625.8	22657.3	22429.6	22218.6	22129.3	22495.8
Log.Lik.	-28035.1	-27593.7	-27436.2	-27278.2	-26808.8	-11318.9	-11205.1	-11089.9	-11040.4	-10800.8
RMSE	0.50	0.49	0.49	0.49	23.550	0.49	0.49	0.49	0.49	
Occupation FE	No	No	No	No	Yes	No	No	No	No	Yes
Birthplace FE	No	No	No	No	Yes	No	No	No	No	Yes

Table SM9*. *Logit model estimates of the likelihood that participants correctly identify a historical figure including control variables. *Each column shows a model with different explanatory variables. Panel A reports log-odds coefficients with standard errors; Panel B shows average marginal effects (AMEs), computed by averaging observation-level marginal effects over each model's estimation sample. For country and language, we present the difference in predicted probabilities between those who share these attributes with the figure and those who do not. Age and gender effects are relative to participants under 40 and male, respectively. For globality, locality, and number of spoken languages, we report the average change in probability per one-unit increase. All models control for the figure's occupation, birthplace, and birth year. Significance: * p < 0.05, ** p < 0.01, *** p < 0.001.*

	**(1)**	**(2)**	**(3)**	**(4)**	**(5)**	**(6)**
**A: (LOG) ODDS RATIO OF CORRECT ANSWER**						
Locality	0.183	0.081	-0.113	0.083	0.095	-0.094
	(0.288)	(0.292)	(0.304)	(0.292)	(0.292)	(0.304)
Globality	0.526	0.305	-0.354	0.302	0.330	-0.317
	(0.714)	(0.721)	(0.748)	(0.721)	(0.721)	(0.749)
Locality × Globality	3.657***	3.947***	4.693***	3.947***	3.917***	4.649***
	(1.086)	(1.094)	(1.132)	(1.094)	(1.095)	(1.133)
Same country		0.379***	0.421***	0.376***	0.377***	0.419***
		(0.066)	(0.069)	(0.066)	(0.066)	(0.069)
Same language		0.203***	0.176**	0.214***	0.202***	0.175*
		(0.061)	(0.065)	(0.065)	(0.061)	(0.069)
Player older than 40			0.549***			0.550***
			(0.039)			(0.039)
Player number of spoken Languages				-0.008		0.001
				(0.016)		(0.017)
Player is a women					-0.098*	-0.112**
					(0.042)	(0.043)
Player declared other gender					-0.069	-0.109
					(0.060)	(0.081)
**B: AVERAGE MARGINAL EFFECTS**						
Locality	0.250***	0.239***	0.234***	0.239***	0.240***	0.235***
	(0.048)	(0.047)	(0.049)	(0.047)	(0.047)	(0.049)
Globality	0.597***	0.571***	0.510***	0.570***	0.572***	0.512***
	(0.039)	(0.039)	(0.040)	(0.039)	(0.039)	(0.040)
Same country		0.084***	0.092***	0.083***	0.083***	0.091***
		(0.014)	(0.015)	(0.014)	(0.014)	(0.015)
Same language		0.046***	0.039**	0.048***	0.046***	0.039*
		(0.014)	(0.014)	(0.015)	(0.014)	(0.015)
Player older than 40			0.123***			0.123***
			(0.009)			(0.009)
Player number of spoken Languages				-0.002		0.000
				(0.004)		(0.004)
Player is a women					-0.022*	-0.025**
					(0.009)	(0.010)
Player declared other gender					-0.016	-0.024
					(0.013)	(0.018)
Num.Obs.	16652	16652	15452	16652	16652	15452
AIC	21785.5	21618.6	19864.1	21620.3	21616.5	19862.2
BIC	22495.8	23070.0	21286.1	23079.4	23083.3	21307.2
Log.Lik.	-10800.766	-10621.281	-9746.032	-10621.154	-10618.243	-9742.109
RMSE	0.48	0.47	0.47	0.47	0.47	0.47
Player nationality FE	No	Yes	Yes	Yes	Yes	Yes

Table SM10. Logit model estimations of the likelihood that participants correctly answer a question about a historical figure, including additional interactions. *Each column reports a different specification, including a subset of the explanatory variables.* ***Panel A:*** *Shows the logit coefficients (indicating changes in log-odds), with odds ratios in square brackets and standard errors in parentheses.* ***Panel B:*** *Presents the estimated average marginal effects for the main variables, computed by averaging observation-level marginal effects over each model's estimation sample. For the "same country" variable, we show the difference in predicted probabilities between participants who share the figure's country of birth and those who do not. For the "global" and "local" fame variables, we report the average change in the estimated probability of answering correctly for a one-unit increase in these scores (dY/dX). Significance levels: * $p < 0.05$, ** $p < 0.01$, *** $p < 0.001$.*

	**(1)**	**(2)**	**(3)**	**(4)**	**(5)**	**(6)**	**(7)**	**(8)**
	**A: (LOG) ODDS RATIO OF CORRECT ANSWER**							
Locality	0.673***	-0.006	-0.118	-0.383	-0.101	-0.399	-0.405	
	(0.147)	(0.176)	(0.304)	(0.308)	(0.304)	(0.312)	(0.312)	
Globality	0.239	0.181	-0.361	-0.519	-0.429	-0.662	-0.653	
	(0.392)	(0.463)	(0.748)	(0.748)	(0.747)	(0.757)	(0.757)	
Locality × Globality	3.246***	4.092***	4.711***	4.995***	4.702***	5.115***	5.102***	
	(0.620)	(0.708)	(1.132)	(1.133)	(1.129)	(1.148)	(1.148)	
Same country			0.468***	-1.099***	0.256*	-1.652*	-1.747*	
			(0.067)	(0.318)	(0.121)	(0.723)	(0.723)	
Locality X Same Country				2.620***		3.143**	3.236**	
				(0.521)		(1.133)	(1.132)	
Globality X Same Country					1.093*	2.904	2.777	
					(0.528)	(3.533)	(3.528)	
Locality × Globality x Same Country						-2.793	-2.692	
						(5.541)	(5.529)	
Same language							0.197**	
							(0.070)	
	**B: AVERAGE MARGINAL EFFECTS**							
Locality	0.343***	0.222***	0.234***	0.248***	0.237***	0.250***	0.251***	
	(0.020)	(0.031)	(0.049)	(0.049)	(0.049)	(0.049)	(0.049)	
Globality	0.494***	0.581***	0.511***	0.510***	0.519***	0.520***	0.518***	
	(0.019)	(0.026)	(0.040)	(0.040)	(0.040)	(0.040)	(0.040)	
Same country			0.101***	0.081***	0.111***	0.092***	0.080***	
			(0.014)	(0.014)	(0.014)	(0.015)	(0.016)	
Same language							0.044**	
							(0.015)	
Num.Obs.	40636	40636	15452	15452	15452	15452	15452	
AIC	54566.3	53807.6	19870.4	19846.5	19867.9	19845.7	19839.7	
BIC	54609.4	54625.8	21292.5	21276.2	21297.6	21290.7	21292.3	
Log.Lik.	-27278.2	-26808.9	-9749.2	-9736.3	-9746.96	-9733.9	-9729.8	
F	379.892	23.550	7.367	7.400	7.310	7.303	7.302	
RMSE	0.49	0.48	0.47	0.47	0.47	0.47	0.47	
Occupation FE	No	Yes	Yes	Yes	Yes	Yes	Yes	
Birthplace FE	No	Yes	Yes	Yes	Yes	Yes	Yes	
Birth Year Control	Yes	Yes	Yes	Yes	Yes	Yes	Yes	
Player N lang Control	No	No	No	Yes	Yes	Yes	Yes	
Player nationality FE	No	No	No	Yes	Yes	Yes	Yes	

Table SM11. Predicted probability that a participant answers a question about a historical figure correctly. *Estimated with Model 6 (table 1) from the main text. Rows combine (i) whether the participant and the figure share the same* ***country*** *and/or* ***language****, and (ii) two levels of Globality and Locality (their means = 0.266 and 0.573; their means + 1 SD = 0.435 and 0.702, respectively). All remaining variables are fixed at their reference values—means for numeric quantities and modes for categorical ones. Player-level references are age band = 20 – 29 and country = United States. Biography-level references are birth year = 1812.99 and occupation = writer, while the biography's country shifts to create the three comparison conditions: when* ***country and language are the same****, both participant and biography are from the United States (English); when* ***countries differ but language is the same****, the biography is set to the United Kingdom (English) and the participant remains in the United States; when* ***both country and language differ****, the biography is set to France (French) and the participant remains in the United States. For each scenario the table reports the predicted probability, its standard error (S. E.), the two-tailed p-value (Pr(>| z |)), and the 95 % confidence interval ("2.5 %" and "97.5 %" columns).*

Country Comparison	Language Comparison	Globality	Locality	Predicted probability	Pr(>|z|)	S.E.	2.5 %	97.5 %
Same	Same	0.266	0.573	0.616	<0.001	29.5	0.579	0.652
Same	Same	0.266	0.702	0.650	<0.001	47.9	0.614	0.685
Same	Same	0.435	0.573	0.705	<0.001	74.3	0.668	0.739
Same	Same	0.435	0.702	0.754	<0.001	137.0	0.723	0.783
Different	Same	0.266	0.573	0.492	0.656	0.6	0.457	0.527
Different	Same	0.266	0.702	0.526	0.163	2.6	0.489	0.562
Different	Same	0.435	0.573	0.588	<0.001	17.3	0.550	0.625
Different	Same	0.435	0.702	0.646	<0.001	49.8	0.612	0.679
Different	Different	0.266	0.573	0.445	0.00101	10.0	0.413	0.478
Different	Different	0.266	0.702	0.479	0.22978	2.1	0.444	0.513
Different	Different	0.435	0.573	0.541	0.02670	5.2	0.505	0.578
Different	Different	0.435	0.702	0.602	< 0.001	26.6	0.567	0.635

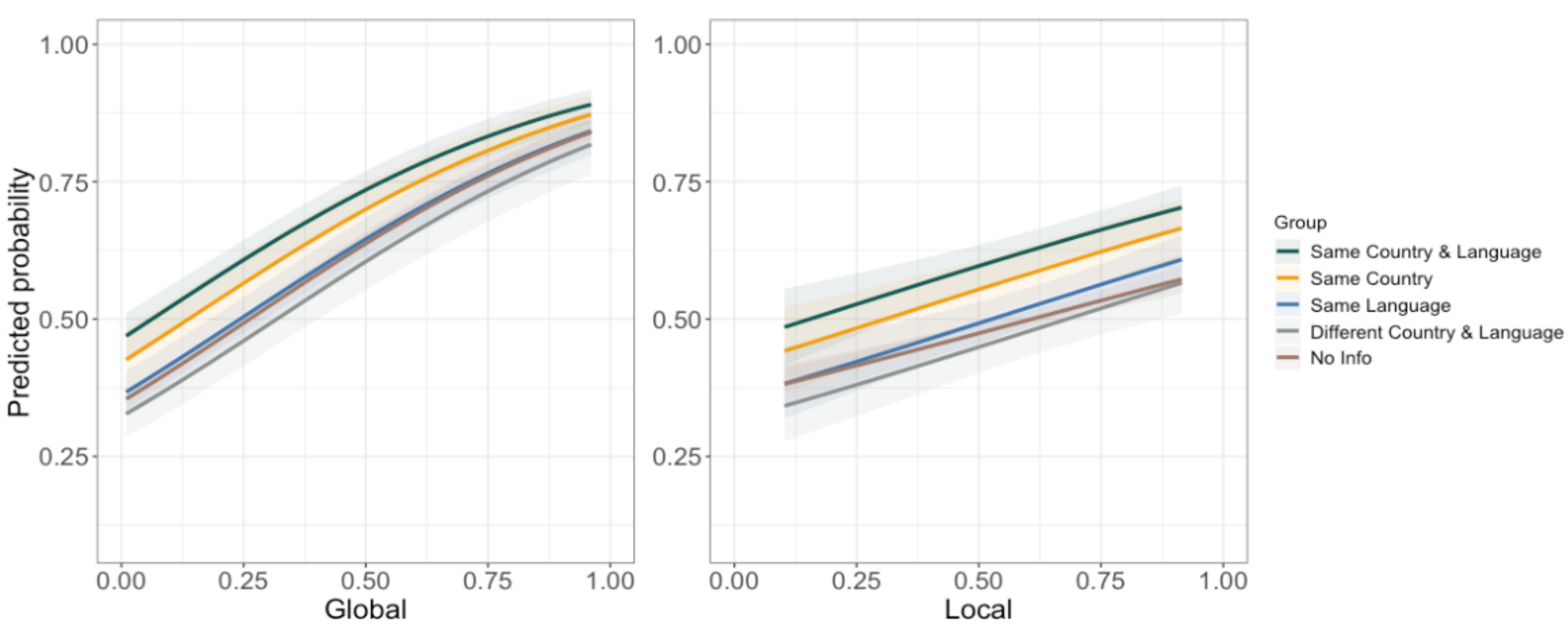

Figure SM8: Predicted probability of correct answers comparing the birthplace of players and the biographies of the historical figures in the questions.

Event study

Now, we analyze how local and global collective memory metrics as well as overall pageviews evolve over time in response to major life events, specifically deaths, Nobel Prize wins, and Oscar awards. To do this, we use our observational dataset of 51,465 historical figures spanning 2015 to 2024. From this dataset, we construct three independent subsets of individuals who (i) died, (ii) received a Nobel Prize, or (iii) won an Oscar during this period. These subsets capture multiple years of observations per figure:

- 1,829 historical figures who died:17,952 biography-year observations.
- 89 Nobel Prize recipients: 853 biography-year observations.
- 46 Oscar winners: 456 biography-year observations.

Each observation represents a biography-year pair, tracking how collective memory metrics evolve. Figure SM9 illustrates the distinct trajectories within our sample, highlighting which years are observed as *before* and *after* periods, as well as any gaps in data across these subsets.

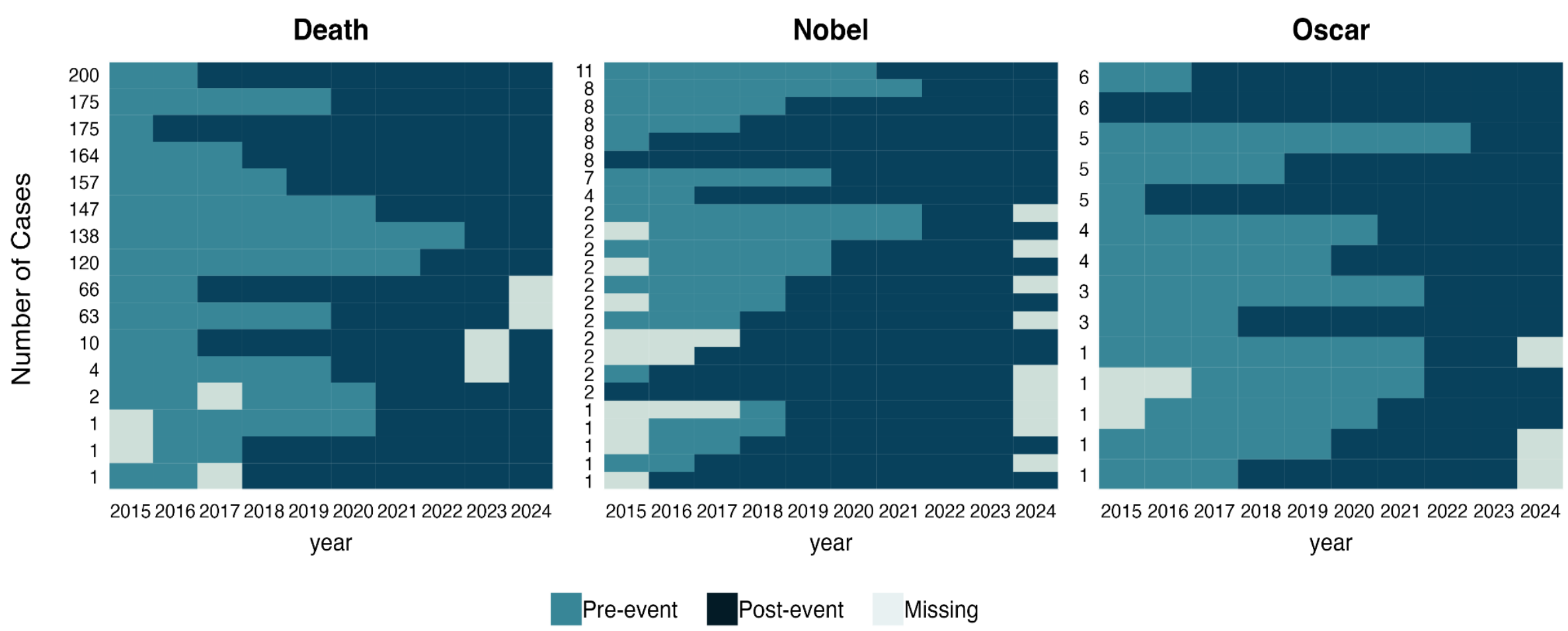

Figure SM9: Unique event histories for Death, Nobel, and Oscar. *Indicates the number of cases in the sample that follow a specific treatment history. For Death, it presents the 15 trajectories with more cases, out of 32 observed in the data.*

To assess these temporal dynamics, we define a set of binary indicators that encode the relative time (k) to the event for each figure *i* in year *t*, allowing for comparisons across figures at the same event timing:

$$Event(t = k) = \begin{cases} 1, & t - t_i^{event} = k \\ 0, & otherwise \end{cases} \tag{4}$$

The year of the event is therefore (k = 0), and the year immediately preceding the event (k = -1) is the baseline; omitted in the regressions. For example, for a Nobel Prize awarded in 2020 (*k = 0*), the relative time indicators are constructed as follows:

• *k = -2* → Two years before (2018)

• *k = -1* → One year before (2019) → Baseline year

• *k = 0* → Event year (2020)

• *k = +1* → One year after (2021)

• *k = +2* → Two years after (2022)

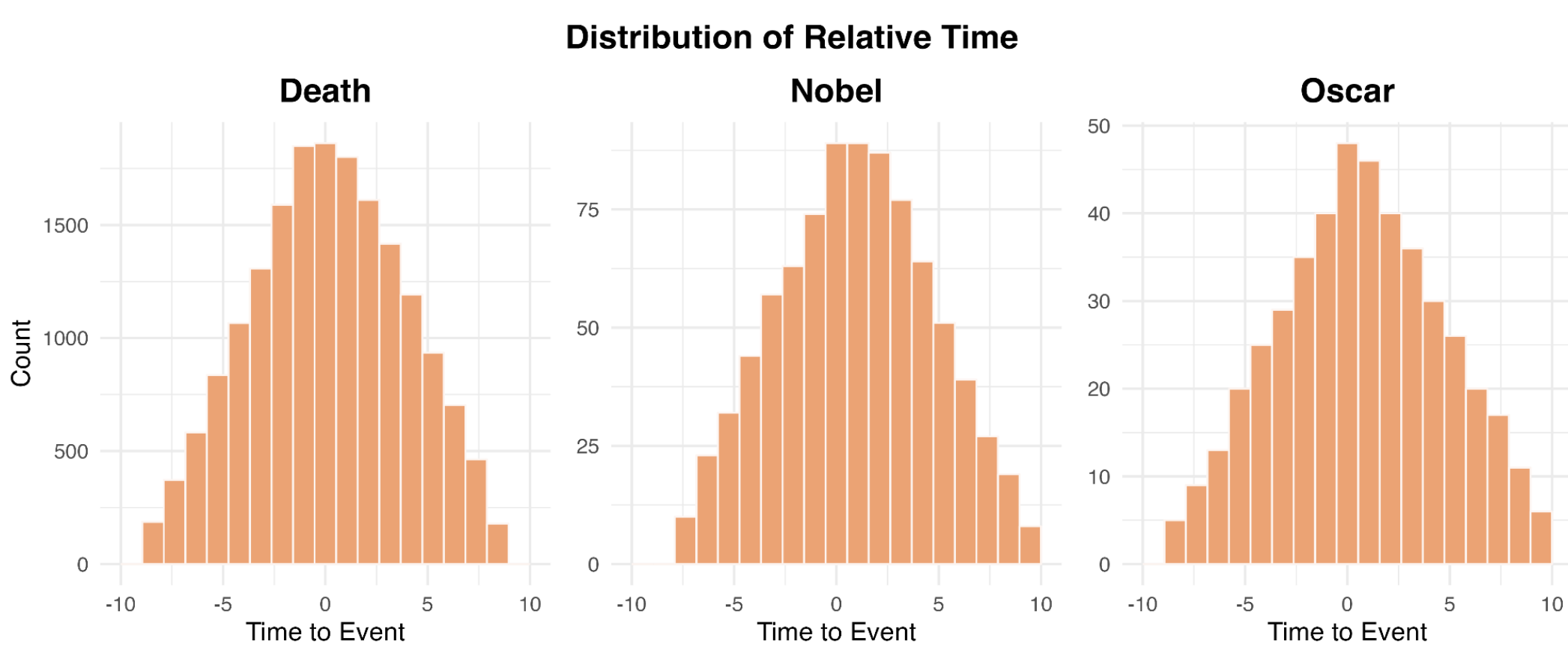

Figure SM10: Distribution of relative time for subsamples used for the event analyses.

To ensure robust data coverage and statistical power, we define a five-year window before and after the event ($|k| \leq 5$), see Figure SM10 to check the distribution across the relative time. Observations beyond this range ($k \leq -5$ or $k \geq +5$) are grouped into broader categories. For instance, any event occurring at k=-6 is included in the k=-5 category. This approach not only preserves

sample size but also avoids perfect multicollinearity with time-fixed effects, as all figures in the sample eventually experience an event.

Using these structured datasets, we model changes in the collective memory outcomes with this following specification:

$$\log(Y_{it}) = \sum_{\substack{k=-5 \\ k\neq -1}}^{5} \beta_k \times Event(t = k) + \alpha_i + \lambda_t + \mu_{it} \tag{5}$$

Where Y_{it} represents the collective memory outcome, measured as overall pageviews, locality (C_{it}), or globality (G_{it}), and is log-transformed for interpretability. $Event(t = k)$ are the relative time dummies, therefore each β_k captures the difference in outcomes for relative year k and the baseline year. α_{it} are individual fixed effects to control for time-invariant characteristics of each historical figure, and λ_{it} are time-fixed effects to account for broader temporal trends affecting all individuals. ϵ_{it} is the error term.

In the manuscript, Figure 4 presents point estimates from our main models, which use a five-year time window and a log-transformed dependent variable; the corresponding numerical results are shown in Table SM12. To test the robustness of our findings, we examine several extended model specifications. Table SM13 adds a control for the number of Wikipedia language editions. Tables SM14 and SM15 replicate the analyses without the log transformation, both excluding and including the language control. Finally, Tables SM16 and SM17 assess sensitivity to expanding the time window to six and seven years, respectively. Overall, the results remain robust across model variations, time frames, and additional controls.

Table SM12: Event study, logarithm transformation in the dependent variable. Estimated staggered effects (Two-Way Fixed Effects, TWFE) on the logarithmic transformation of the dependent variables relative to the event's timing. The percentual changes in the dependent variable are shown in brackets, calculated as $(e\widehat{(\beta_k)} - 1) \times 100$. The models incorporate individual and time-fixed effects. Standard errors are clustered at the individual and year level. Statistical significance levels: + p < 0.1, * p < 0.05, ** p < 0.01, *** p < 0.001.

**Dep.Var**	**Log(PageViews)**			**Log(Locality)**			**Log(Globality)**		
**Event**	**Death**	**Nobel**	**Oscar**	**Death**	**Nobel**	**Oscar**	**Death**	**Nobel**	**Oscar**
**Relative Time**									
5 years<br>before<br>or more	-0.003<br>[-0.3%]<br>p = 0.928<br>SE= 0.035	-0.326<br>[-27.8%]<br>p = 0.109<br>SE= 0.181	-0.438<br>[-35.5%]*<br>p = 0.03<br>SE= 0.166	-0.006<br>[-0.6%]<br>p = 0.453<br>SE= 0.008	-0.183<br>[-16.7%]*<br>p = 0.014<br>SE= 0.058	-0.025<br>[-2.5%]<br>p = 0.575<br>SE= 0.043	-0.021<br>[-2.1%]<br>p = 0.358<br>SE= 0.022	-0.361<br>[-30.3%]*<br>p = 0.044<br>SE= 0.151	0.163<br>[17.7%]<br>p = 0.422<br>SE= 0.192
4 years<br>before	-0.018<br>[-1.7%]<br>p = 0.566<br>SE= 0.029	-0.147<br>[-13.6%]*<br>p = 0.039<br>SE= 0.059	-0.277<br>[-24.2%]<br>p = 0.091<br>SE= 0.144	-0.013<br>[-1.3%]<br>p = 0.181<br>SE= 0.009	-0.127<br>[-12%]*<br>p = 0.048<br>SE= 0.055	-0.002<br>[-0.2%]<br>p = 0.959<br>SE= 0.043	-0.03<br>[-3%]<br>p = 0.112<br>SE= 0.017	-0.281<br>[-24.5%]*<br>p = 0.031<br>SE= 0.108	0.026<br>[2.6%]<br>p = 0.874<br>SE= 0.158
3 years<br>before	-0.023<br>[-2.3%]<br>p = 0.179<br>SE= 0.015	-0.125<br>[-11.7%]<br>p = 0.135<br>SE= 0.075	-0.184<br>[-16.8%]<br>p = 0.052<br>SE= 0.081	-0.008<br>[-0.8%]<br>p = 0.366<br>SE= 0.008	-0.08<br>[-7.7%]*<br>p = 0.031<br>SE= 0.03	-0.011<br>[-1.1%]<br>p = 0.179<br>SE= 0.008	-0.018<br>[-1.7%]<br>p = 0.186<br>SE= 0.012	-0.073<br>[-7%]*<br>p = 0.024<br>SE= 0.026	0.125<br>[13.3%]<br>p = 0.364<br>SE= 0.13
2 years<br>before	-0.004<br>[-0.4%]<br>p = 0.742<br>SE= 0.012	0.021<br>[2.1%]<br>p = 0.741<br>SE= 0.06	-0.096<br>[-9.2%]<br>p = 0.365<br>SE= 0.1	0.001<br>[0.1%]<br>p = 0.863<br>SE= 0.005	-0.003<br>[-0.3%]<br>p = 0.195<br>SE= 0.002	0.015<br>[1.5%]<br>p = 0.545<br>SE= 0.024	-0.017<br>[-1.7%]*<br>p = 0.043<br>SE= 0.007	-0.064<br>[-6.2%]<br>p = 0.218<br>SE= 0.048	0.1<br>[10.6%]<br>p = 0.107<br>SE= 0.055
**Event<br>year**	**1.034<br>[181.1%]***<br>p = <0.001<br>SE= 0.018**	**2.687<br>[1369.1%]***<br>p = <0.001<br>SE= 0.157**	**0.515<br>[67.3%]***<br>p = <0.001<br>SE= 0.066**	**0.204<br>[22.6%]***<br>p = <0.001<br>SE= 0.009**	**0.596<br>[81.6%]***<br>p = <0.001<br>SE= 0.042**	**0.073<br>[7.5%]**<br>p = 0.003<br>SE= 0.018**	**0.115<br>[12.2%]*<br>p = 0.014<br>SE= 0.037**	**1.269<br>[255.6%]***<br>p = <0.001<br>SE= 0.162**	**-0.125<br>[-11.7%]<br>p = 0.179<br>SE= 0.085**
1 years<br>after	0.157<br>[17%]***<br>p = <0.001<br>SE= 0.019	2.158<br>[765%]***<br>p = <0.001<br>SE= 0.124	0.74<br>[109.7%]**<br>p = 0.001<br>SE= 0.149	0.021<br>[2.1%]*<br>p = 0.023<br>SE= 0.007	0.466<br>[59.4%]***<br>p = <0.001<br>SE= 0.035	0.105<br>[11%]**<br>p = 0.004<br>SE= 0.026	0.34<br>[40.5%]***<br>p = <0.001<br>SE= 0.035	1.722<br>[459.7%]***<br>p = <0.001<br>SE= 0.158	0.132<br>[14.1%]<br>p = 0.407<br>SE= 0.15
2 years<br>after	0.063<br>[6.5%]**<br>p = 0.01<br>SE= 0.019	1.887<br>[559.9%]***<br>p = <0.001<br>SE= 0.15	0.317<br>[37.4%]*<br>p = 0.027<br>SE= 0.117	0.006<br>[0.6%]<br>p = 0.616<br>SE= 0.011	0.4<br>[49.2%]***<br>p = <0.001<br>SE= 0.053	0.047<br>[4.8%]*<br>p = 0.021<br>SE= 0.016	0.362<br>[43.6%]***<br>p = <0.001<br>SE= 0.035	1.861<br>[543.2%]***<br>p = <0.001<br>SE= 0.179	0.496<br>[64.2%]*<br>p = 0.014<br>SE= 0.158
3 years<br>after	0.051<br>[5.2%]<br>p = 0.121<br>SE= 0.029	1.735<br>[466.7%]***<br>p = <0.001<br>SE= 0.144	0.346<br>[41.4%]*<br>p = 0.035<br>SE= 0.136	0.001<br>[0.1%]<br>p = 0.939<br>SE= 0.011	0.385<br>[47%]***<br>p = <0.001<br>SE= 0.046	0.047<br>[4.8%]<br>p = 0.082<br>SE= 0.024	0.377<br>[45.9%]***<br>p = <0.001<br>SE= 0.046	1.925<br>[585.2%]***<br>p = <0.001<br>SE= 0.198	0.562<br>[75.4%]*<br>p = 0.034<br>SE= 0.22
4 years<br>after	0.024<br>[2.4%]<br>p = 0.483<br>SE= 0.032	1.693<br>[443.8%]***<br>p = <0.001<br>SE= 0.173	0.173<br>[18.9%]<br>p = 0.208<br>SE= 0.126	-0.005<br>[-0.5%]<br>p = 0.772<br>SE= 0.016	0.363<br>[43.7%]***<br>p = <0.001<br>SE= 0.068	0.029<br>[2.9%]<br>p = 0.165<br>SE= 0.019	0.388<br>[47.4%]***<br>p = <0.001<br>SE= 0.058	2.01<br>[646.7%]***<br>p = <0.001<br>SE= 0.227	0.623<br>[86.4%]<br>p = 0.055<br>SE= 0.278
5 years<br>after or<br>more	0.015<br>[1.5%]<br>p = 0.693<br>SE= 0.037	1.699<br>[446.9%]***<br>p = <0.001<br>SE= 0.192	0.164<br>[17.9%]<br>p = 0.261<br>SE= 0.136	-0.006<br>[-0.6%]<br>p = 0.701<br>SE= 0.015	0.422<br>[52.5%]***<br>p = <0.001<br>SE= 0.08	0.028<br>[2.9%]<br>p = 0.339<br>SE= 0.028	0.408<br>[50.4%]***<br>p = <0.001<br>SE= 0.073	2.1<br>[716.3%]***<br>p = <0.001<br>SE= 0.3	0.654<br>[92.4%]<br>p = 0.118<br>SE= 0.374
Num.Obs.	16377	675	397	16377	675	397	16225	664	384
R2	0.967	0.931	0.913	0.888	0.864	0.843	0.870	0.893	0.861
R2 Adj.	0.963	0.920	0.897	0.874	0.842	0.815	0.853	0.875	0.835
FE Bio.	1829	75	44	1829	75	44	1828	75	44
FE Years	9	9	9	9	9	9	9	9	9

Table SM13: Event study, logarithm transformation, controlling for number of languages. Estimated staggered effects (Two-Way Fixed Effects, TWFE) on the logarithmic transformation of the dependent variables relative to the event's timing. The percentual changes in the dependent variable are shown in brackets, calculated as $(e(\widehat{\beta_k}) - 1) \times 100$.The models incorporate individual and time-fixed effects. Standard errors are clustered at the individual and year level. Statistical significance levels: + p < 0.1, * p < 0.05, ** p < 0.01, *** p < 0.001.

**Dep.Var**	**Log(PageViews)**			**Log(Locality)**			**Log(Globality)**		
**Event**	**Death**	**Nobel**	**Oscar**	**Death**	**Nobel**	**Oscar**	**Death**	**Nobel**	**Oscar**
**Relative Time**									
5 years	-0.007	-0.325	-0.344	-0.007	-0.182	-0.015	-0.007	-0.385	0.06
before	[-0.7%]	[-27.7%]	[-29.1%]*	[-0.7%]	[-16.6%]*	[-1.4%]	[-0.7%]	[-31.9%]*	[6.2%]
or more	p = 0.838	p = 0.114	p = 0.012	p = 0.394	p = 0.013	p = 0.716	p = 0.663	p = 0.014	p = 0.772
4 years	-0.019	-0.146	-0.284	-0.014	-0.127	-0.003	-0.024	-0.287	0.047
before	[-1.9%]	[-13.6%]	[-24.7%]	[-1.3%]	[-11.9%]*	[-0.3%]	[-2.3%]	[-25%]**	[4.8%]
	p = 0.519	p = 0.059	p = 0.056	p = 0.167	p = 0.047	p = 0.945	p = 0.187	p = 0.004	p = 0.748
3 years	-0.025	-0.124	-0.141	-0.009	-0.08	-0.007	-0.01	-0.087	0.083
before	[-2.5%]	[-11.7%]	[-13.1%]	[-0.9%]	[-7.7%]*	[-0.6%]	[-1%]	[-8.3%]***	[8.7%]
	p = 0.148	p = 0.132	p = 0.185	p = 0.322	p = 0.029	p = 0.322	p = 0.254	p = <0.001	p = 0.521
2 years	-0.005	0.022	-0.032	0.001	-0.002	0.022	-0.012	-0.074	0.042
before	[-0.5%]	[2.2%]	[-3.1%]	[0.1%]	[-0.2%]	[2.3%]	[-1.2%]	[-7.1%]	[4.3%]
	p = 0.685	p = 0.679	p = 0.756	p = 0.913	p = 0.332	p = 0.368	p = 0.138	p = 0.107	p = 0.592
**Event**	**1.045**	**2.722**	**0.387**	**0.206**	**0.607**	**0.059**	**0.072**	**0.888**	**0.039**
**year**	**[184.4%]*** **	**[1421.2%]*** **	**[47.3%]*** **	**[22.9%]*** **	**[83.5%]*** **	**[6%]** **	**[7.5%]***	**[142.9%]*** **	**[4%]**
	**p = <0.001**	**p = <0.001**	**p = <0.001**	**p = <0.001**	**p = <0.001**	**p = 0.002**	**p = 0.014**	**p = <0.001**	**p = 0.543**
1 years	0.207	2.225	0.822	0.033	0.487	0.114	0.155	0.989	0.05
after	[23%]***	[825.2%]***	[127.5%]***	[3.4%]**	[62.8%]***	[12.1%]**	[16.8%]***	[168.9%]***	[5.2%]
	p = <0.001	p = <0.001	p = <0.001	p = 0.002	p = <0.001	p = 0.003	p = <0.001	p = <0.001	p = 0.668
2 years	0.116	1.962	0.492	0.019	0.424	0.067	0.169	1.043	0.322
after	[12.2%]**	[611.4%]***	[63.6%]**	[1.9%]	[52.8%]***	[6.9%]*	[18.4%]***	[183.7%]***	[38%]*
	p = 0.002	p = <0.001	p = 0.007	p = 0.135	p = <0.001	p = 0.018	p = <0.001	p = <0.001	p = 0.043
3 years	0.104	1.814	0.51	0.014	0.41	0.065	0.182	1.058	0.393
after	[10.9%]*	[513.6%]**	[66.5%]**	[1.4%]	[50.7%]***	[6.7%]*	[20%]***	[187.9%]***	[48.1%]
	p = 0.033	p = 0.001	p = 0.004	p = 0.297	p = <0.001	p = 0.036	p = <0.001	p = <0.001	p = 0.079
4 years	0.079	1.773	0.384	0.008	0.387	0.053	0.186	1.146	0.415
after	[8.2%]	[488.7%]***	[46.9%]*	[0.8%]	[47.3%]***	[5.4%]	[20.5%]**	[214.6%]***	[51.4%]
	p = 0.1	p = <0.001	p = 0.023	p = 0.655	p = <0.001	p = 0.074	p = 0.002	p = <0.001	p = 0.115
5 years	0.074	1.779	0.456	0.009	0.447	0.06	0.19	1.227	0.359
after or	[7.7%]	[492.6%]***	[57.8%]*	[0.9%]	[56.3%]***	[6.2%]	[20.9%]**	[240.9%]**	[43.2%]
more	p = 0.183	p = <0.001	p = 0.029	p = 0.628	p = <0.001	p = 0.144	p = 0.003	p = 0.001	p = 0.319
Number of	-0.014	-0.003	-0.052	-0.003	-0.001	-0.006	0.051	0.03	0.056
languages	[-1.4%]*	[-0.3%]	[-5%]***	[-0.3%]**	[-0.1%]	[-0.6%]**	[5.3%]***	[3%]***	[5.7%]***
	p = 0.019	p = 0.826	p = <0.001	p = 0.006	p = 0.617	p = 0.001	p = <0.001	p = <0.001	p = <0.001
Num.Obs.	16377	675	397	16377	675	397	16225	664	384
R2	0.968	0.931	0.928	0.888	0.864	0.857	0.913	0.912	0.910
R2 Adj.	0.964	0.920	0.915	0.874	0.842	0.830	0.902	0.897	0.893
FE Bio.	1829	75	44	1829	75	44	1828	75	44
FE Years	9	9	9	9	9	9	9	9	9

Table SM14: Event study with raw dependent variables. Estimated staggered effects (Two-Way Fixed Effects, TWFE) relative to the event's timing. The models incorporate individual and time-fixed effects. Standard errors are clustered at the individual and year level. Statistical significance levels: * p < 0.05, ** p < 0.01, *** p < 0.001.

**Dep .Var.**	**PageViews**			**Locality**			**Globality**		
**Event**	**Death**	**Nobel**	**Oscar**	**Death**	**Nobel**	**Oscar**	**Death**	**Nobel**	**Oscar**
**Rel. Time**									
5 years before or more	107179.46**<br>SE= 24759.75<br>p = 0.003	488049.46<br>SE= 385228.28<br>p = 0.241	-712246.18<br>SE= 1485708.1<br>p = 0.644	0.004<br>SE= 0.003<br>p = 0.213	-0.081*<br>SE= 0.034<br>p = 0.044	-0.021<br>SE= 0.026<br>p = 0.439	-0.002<br>SE= 0.001<br>p = 0.157	-0.013<br>SE= 0.008<br>p = 0.151	0.004<br>SE= 0.015<br>p = 0.775
4 years before	122746.65*<br>SE= 37527.4<br>p = 0.011	386163.38<br>SE= 303412.85<br>p = 0.239	-540878.37<br>SE= 1070578.01<br>p = 0.627	0.001<br>SE= 0.003<br>p = 0.855	-0.055*<br>SE= 0.024<br>p = 0.049	-0.007<br>SE= 0.025<br>p = 0.79	-0.002<br>SE= 0.001<br>p = 0.166	-0.01<br>SE= 0.006<br>p = 0.164	-0.004<br>SE= 0.01<br>p = 0.691
3 years before	62784.82*<br>SE= 22534.74<br>p = 0.024	230885.65<br>SE= 148316.60<br>p = 0.158	-155935.48<br>SE= 990024.25<br>p = 0.879	0.0001<br>SE= 0.003<br>p = 0.957	-0.039*<br>SE= 0.015<br>p = 0.033	-0.012**<br>SE= 0.004<br>p = 0.008	-0.001<br>SE= 0.001<br>p = 0.148	-0.004<br>SE= 0.003<br>p = 0.213	0.002<br>SE= 0.007<br>p = 0.816
2 years before	56481.30*<br>SE= 23074.43<br>p = 0.04	113878.63*<br>SE= 49382.39<br>p = 0.05	-446070.33<br>SE= 788705.63<br>p = 0.587	0.003<br>SE= 0.002<br>p = 0.145	-0.004<br>SE= 0.003<br>p = 0.174	0.002<br>SE= 0.011<br>p = 0.838	-0.001<br>SE= 0.001<br>p = 0.231	-0.001<br>SE= 0.003<br>p = 0.777	0.004<br>SE= 0.003<br>p = 0.251
**Event year**	771498.8***<br>SE= 73367.47<br>p = <0.001	556157.93**<br>SE= 146110.88<br>p = 0.005	1974241.54**<br>SE= 417363.82<br>p = 0.001	0.092***<br>SE= 0.003<br>p = <0.001	0.239***<br>SE= 0.015<br>p = <0.001	0.046***<br>SE= 0.008<br>p = <0.001	0.005*<br>SE= 0.001<br>p = 0.011	0.068***<br>SE= 0.008<br>p = <0.001	-0.01<br>SE= 0.006<br>p = 0.11
1 years after	-8064.55<br>SE= 54019.51<br>p = 0.885	210022.46<br>SE= 106875.4<br>p = 0.085	971644.85<br>SE= 597171.23<br>p = 0.142	0.005<br>SE= 0.003<br>p = 0.09	0.169***<br>SE= 0.012<br>p = <0.001	0.062**<br>SE= 0.013<br>p = 0.001	0.018***<br>SE= 0.002<br>p = <0.001	0.128***<br>SE= 0.006<br>p = <0.001	0.011<br>SE= 0.01<br>p = 0.308
2 years after	-87309.27<br>SE= 54059.09<br>p = 0.145	315864.61*<br>SE= 125955.1<br>p = 0.036	-226309.31<br>SE= 568168.83<br>p = 0.701	-0.003<br>SE= 0.004<br>p = 0.393	0.14***<br>SE= 0.024<br>p = <0.001	0.025*<br>SE= 0.008<br>p = 0.013	0.019***<br>SE= 0.002<br>p = <0.001	0.144***<br>SE= 0.009<br>p = <0.001	0.021<br>SE= 0.012<br>p = 0.121
3 years after	-138256.48<br>SE= 61270.95<br>p = 0.054	91339.26<br>SE= 75652.65<br>p = 0.262	-103007.699<br>SE= 780862.93<br>p = 0.898	-0.006<br>SE= 0.004<br>p = 0.17	0.133***<br>SE= 0.025<br>p = <0.001	0.025<br>SE= 0.013<br>p = 0.098	0.02***<br>SE= 0.002<br>p = <0.001	0.151***<br>SE= 0.011<br>p = <0.001	0.022<br>SE= 0.015<br>p = 0.176
4 years after	-207545.14*<br>SE= 78496.71<br>p = 0.03	-128347.87<br>SE= 288906.54<br>p = 0.669	-660280.21<br>SE= 971160.99<br>p = 0.516	-0.01<br>SE= 0.005<br>p = 0.084	0.12*<br>SE= 0.04<br>p = 0.018	0.014<br>SE= 0.011<br>p = 0.213	0.021***<br>SE= 0.003<br>p = <0.001	0.154***<br>SE= 0.011<br>p = <0.001	0.027<br>SE= 0.019<br>p = 0.2
5 years after or more	-292838.9*<br>SE= 104959.22<br>p = 0.024	-227782.75<br>SE= 388278.004<br>p = 0.574	-997317.1<br>SE= 1190209.4<br>p = 0.426	-0.013*<br>SE= 0.005<br>p = 0.035	0.153**<br>SE= 0.045<br>p = 0.009	0.017<br>SE= 0.015<br>p = 0.295	0.022***<br>SE= 0.003<br>p = <0.001	0.159***<br>SE= 0.013<br>p = <0.001	0.039<br>SE= 0.025<br>p = 0.159
Num.Obs.	16377	675	397	16377	675	397	16377	675	397
R2	0.684	0.598	0.811	0.898	0.886	0.852	0.940	0.932	0.925
R2 Adj.	0.644	0.535	0.776	0.885	0.867	0.825	0.933	0.921	0.911
FE Bio.	1829	75	44	1829	75	44	1829	75	44
FE Years	9	9	9	9	9	9	9	9	9

Table SM15: Event study with raw dependent variables, controlling for the number of languages. Estimated staggered effects (Two-Way Fixed Effects, TWFE) relative to the event's timing. The models incorporate individual and time-fixed effects. Standard errors are clustered at the individual and year level. Statistical significance levels: + p < 0.1, * p < 0.05, ** p < 0.01, *** p < 0.001.

**Dep .Var.**	**PageViews**			**Locality**			**Globality**		
**Event**	**Death**	**Nobel**	**Oscar**	**Death**	**Nobel**	**Oscar**	**Death**	**Nobel**	**Oscar**
**Relative Time**									
5 years before or more	86561.5***<br>SE= 14144.4<br>p = <0.001	510381.5<br>SE= 394889.5<br>p = 0.232	-373859.1<br>SE= 1090731.7<br>p = 0.741	0.0037<br>SE= 0.003<br>p = 0.315	-0.0807*<br>SE= 0.034<br>p = 0.044	-0.0145<br>SE= 0.023<br>p = 0.543	-0.0008<br>SE= 0.001<br>p = 0.33	-0.0160*<br>SE= 0.005<br>p = 0.013	-0.0041<br>SE= 0.004<br>p = 0.39
4 years before	113173.9*<br>SE= 46481.0<br>p = 0.041	387944.4<br>SE= 297628.8<br>p = 0.229	-567189.0<br>SE= 957870.5<br>p = 0.57	0.0002<br>SE= 0.003<br>p = 0.939	-0.0553*<br>SE= 0.023<br>p = 0.045	-0.0075<br>SE= 0.026<br>p = 0.78	-0.0009<br>SE= 0.001<br>p = 0.153	-0.0101*<br>SE= 0.004<br>p = 0.022	-0.0034<br>SE= 0.003<br>p = 0.316
3 years before	49852.1***<br>SE= 2946.5<br>p = <0.001	242483.2<br>SE= 158743.4<br>p = 0.165	1669.4<br>SE= 925230.8<br>p = 0.999	-0.0003<br>SE= 0.003<br>p = 0.932	-0.0386*<br>SE= 0.015<br>p = 0.031	-0.0092**<br>SE= 0.003<br>p = 0.008	-0.0007<br>SE= 0.000<br>p = 0.094	-0.0056*<br>SE= 0.002<br>p = 0.036	-0.0023<br>SE= 0.003<br>p = 0.425
2 years before	49398.7<br>SE= 25457.5<br>p = 0.088	126911.3<br>SE= 63668.3<br>p = 0.081	-213594.4<br>SE= 683294.5<br>p = 0.763	0.0024<br>SE= 0.002<br>p = 0.254	-0.0036<br>SE= 0.002<br>p = 0.118	0.0071<br>SE= 0.011<br>p = 0.544	-0.0003<br>SE= 0.000<br>p = 0.145	-0.0025<br>SE= 0.001<br>p = 0.064	-0.0017<br>SE= 0.001<br>p = 0.204
**Event year**	**832305.3*****<br>**SE= 68214.9**<br>**p = <0.001**	**1026853.3***<br>**SE= 369612.4**<br>**p = 0.024**	**1515428.8****<br>**SE= 388117.9**<br>**p = 0.005**	**0.0945*****<br>**SE= 0.004**<br>**p = <0.001**	**0.2540*****<br>**SE= 0.018**<br>**p = <0.001**	**0.0367*****<br>**SE= 0.006**<br>**p = <0.001**	**0.0010***<br>**SE= 0.000**<br>**p = 0.048**	**0.0075**<br>**SE= 0.003**<br>**p = 0.064**	**0.0012**<br>**SE= 0.001**<br>**p = 0.08**
1 years after	250828.4*<br>SE= 77451.9<br>p = 0.012	1116733.4<br>SE= 611563.2<br>p = 0.105	1264447.9*<br>SE= 416465.4<br>p = 0.016	0.0146**<br>SE= 0.003<br>p = 0.001	0.1985***<br>SE= 0.019<br>p = <0.001	0.0681***<br>SE= 0.013<br>p = <0.001	0.0021*<br>SE= 0.001<br>p = 0.048	0.0119<br>SE= 0.006<br>p = 0.072	0.0040<br>SE= 0.002<br>p = 0.088
2 years after	184226.5<br>SE= 85051.8<br>p = 0.062	1329327.5<br>SE= 917211.2<br>p = 0.185	400979.8<br>SE= 371444.8<br>p = 0.312	0.0063<br>SE= 0.004<br>p = 0.126	0.1732***<br>SE= 0.026<br>p = <0.001	0.0375**<br>SE= 0.011<br>p = 0.007	0.0024*<br>SE= 0.001<br>p = 0.035	0.0142<br>SE= 0.008<br>p = 0.101	0.0049<br>SE= 0.002<br>p = 0.08
3 years after	135922.3<br>SE= 90267.4<br>p = 0.171	1164187.2<br>SE= 765980.4<br>p = 0.167	485362.4<br>SE= 460996.6<br>p = 0.323	0.0041<br>SE= 0.005<br>p = 0.4	0.1680***<br>SE= 0.028<br>p = <0.001	0.0366*<br>SE= 0.013<br>p = 0.025	0.0028*<br>SE= 0.001<br>p = 0.04	0.0136<br>SE= 0.008<br>p = 0.11	0.0075<br>SE= 0.003<br>p = 0.062
4 years after	77405.7<br>SE= 99589.2<br>p = 0.459	940886.3<br>SE= 601723.7<br>p = 0.157	99524.9<br>SE= 592931.0<br>p = 0.871	0.0004<br>SE= 0.006<br>p = 0.945	0.1550**<br>SE= 0.036<br>p = 0.003	0.0296*<br>SE= 0.013<br>p = 0.049	0.0034*<br>SE= 0.001<br>p = 0.036	0.0168<br>SE= 0.009<br>p = 0.087	0.0077<br>SE= 0.004<br>p = 0.06
5 years after or more	15491.0<br>SE= 117735.6<br>p = 0.899	853716.7<br>SE= 558961.0<br>p = 0.165	50735.2<br>SE= 733401.3<br>p = 0.947	-0.0018<br>SE= 0.006<br>p = 0.779	0.1881**<br>SE= 0.044<br>p = 0.003	0.0382<br>SE= 0.020<br>p = 0.087	0.0031*<br>SE= 0.001<br>p = 0.037	0.0208<br>SE= 0.010<br>p = 0.08	0.0128<br>SE= 0.006<br>p = 0.054
Number of languages	-71737.0*<br>SE= 22965.0<br>p = 0.014	-37130.4<br>SE= 29566.1<br>p = 0.245	-185445.0**<br>SE= 47307.3<br>p = 0.004	-0.0025***<br>SE= 0.000<br>p = <0.001	-0.0012<br>SE= 0.001<br>p = 0.176	-0.0037***<br>SE= 0.001<br>p = <0.001	0.0044***<br>SE= 0.000<br>p = <0.001	0.0048***<br>SE= 0.000<br>p = <0.001	0.0046***<br>SE= 0.000<br>p = <0.001
Num.Obs.	16377	675	397	16377	675	397	16377	675	397
R2	0.695	0.621	0.837	0.900	0.887	0.868	0.993	0.997	0.993
R2 Adj.	0.657	0.561	0.806	0.887	0.869	0.843	0.992	0.996	0.991
FE Bio.	1829	75	44	1829	75	44	1829	75	44
FE Years	9	9	9	9	9	9	9	9	9

Table SM16: Event study, with a 6-year time window. Estimated staggered effects (Two-Way Fixed Effects, TWFE) relative to the event's timing. The models incorporate individual and time-fixed effects. The percentual changes in the dependent variable are shown in brackets, calculated as $(e\widehat{(\beta_k)} - 1) \times 100$. Standard errors are clustered at the individual and year levels. Statistical significance levels: * $p < 0.05$, ** $p < 0.01$, *** $p < 0.001$

**Dep.Var**	**Log(PageViews)**			**Log(Locality)**			**Log(Globality)**		
**Event**	**Death**	**Nobel**	**Oscar**	**Death**	**Nobel**	**Oscar**	**Death**	**Nobel**	**Oscar**
**Relative Time**									
6 years before or more	0.042<br>[4.3%]<br>p = 0.235	-0.749<br>[-52.7%]<br>p = 0.189	-0.064<br>[-6.2%]<br>p = 0.892	0.016<br>[1.6%]<br>p = 0.359	-0.38<br>[-31.6%]*<br>p = 0.025	-0.014<br>[-1.4%]<br>p = 0.755	-0.047<br>[-4.6%]<br>p = 0.383	-0.332<br>[-28.2%]<br>p = 0.144	0.116<br>[12.3%]<br>p = 0.637
5 years before	0.025<br>[2.5%]<br>p = 0.354	-0.583<br>[-44.2%]<br>p = 0.056	-0.201<br>[-18.2%]<br>p = 0.478	0.007<br>[0.7%]<br>p = 0.597	-0.294<br>[-25.5%]***<br>p = <0.001	-0.016<br>[-1.6%]<br>p = 0.716	-0.047<br>[-4.6%]<br>p = 0.212	-0.426<br>[-34.7%]<br>p = 0.057	0.159<br>[17.3%]<br>p = 0.54
4 years before	0.006<br>[0.6%]<br>p = 0.783	-0.364<br>[-30.5%]<br>p = 0.167	-0.085<br>[-8.1%]<br>p = 0.752	-0.002<br>[-0.2%]<br>p = 0.858	-0.226<br>[-20.2%]**<br>p = 0.009	0.004<br>[0.4%]<br>p = 0.881	-0.046<br>[-4.5%]<br>p = 0.056	-0.295<br>[-25.5%]**<br>p = 0.007	0.008<br>[0.8%]<br>p = 0.966
3 years before	-0.007<br>[-0.7%]<br>p = 0.729	-0.271<br>[-23.7%]<br>p = 0.113	-0.054<br>[-5.3%]<br>p = 0.773	-0.001<br>[-0.1%]<br>p = 0.941	-0.146<br>[-13.6%]**<br>p = 0.007	-0.007<br>[-0.7%]<br>p = 0.52	-0.028<br>[-2.8%]*<br>p = 0.04	-0.083<br>[-7.9%]*<br>p = 0.033	0.113<br>[12%]<br>p = 0.455
2 years before	0.004<br>[0.4%]<br>p = 0.789	-0.053<br>[-5.2%]<br>p = 0.385	-0.032<br>[-3.2%]<br>p = 0.834	0.005<br>[0.5%]<br>p = 0.462	-0.036<br>[-3.5%]<br>p = 0.299	0.017<br>[1.7%]<br>p = 0.439	-0.022<br>[-2.2%]<br>p = 0.114	-0.07<br>[-6.8%]<br>p = 0.208	0.095<br>[10%]<br>p = 0.086
**Event year**	**1.026<br>[179%]***<br>p = <0.001**	**2.759<br>[1478%]***<br>p = <0.001**	**0.452<br>[57.1%]**<br>p = 0.001**	**0.2<br>[22.1%]***<br>p = <0.001**	**0.629<br>[87.5%]***<br>p = <0.001**	**0.071<br>[7.3%]**<br>p = 0.002**	**0.121<br>[12.8%]*<br>p = 0.012**	**1.276<br>[258.2%]***<br>p = <0.001**	**-0.12<br>[-11.3%]<br>p = 0.19**
1 years after	0.142<br>[15.3%]***<br>p = <0.001	2.3<br>[897.7%]***<br>p = <0.001	0.615<br>[85%]*<br>p = 0.015	0.014<br>[1.4%]<br>p = 0.124	0.531<br>[70.1%]***<br>p = <0.001	0.101<br>[10.6%]**<br>p = 0.007	0.35<br>[42%]***<br>p = <0.001	1.734<br>[466%]***<br>p = <0.001	0.143<br>[15.4%]<br>p = 0.385
2 years after	0.04<br>[4.1%]<br>p = 0.152	2.102<br>[718.3%]***<br>p = <0.001	0.128<br>[13.6%]<br>p = 0.529	-0.005<br>[-0.5%]<br>p = 0.645	0.498<br>[64.5%]***<br>p = <0.001	0.041<br>[4.2%]<br>p = 0.136	0.378<br>[45.9%]***<br>p = <0.001	1.876<br>[553%]***<br>p = <0.001	0.513<br>[67.1%]*<br>p = 0.038
3 years after	0.02<br>[2%]<br>p = 0.637	2.022<br>[655.7%]***<br>p = <0.001	0.092<br>[9.6%]<br>p = 0.736	-0.014<br>[-1.4%]<br>p = 0.342	0.515<br>[67.4%]***<br>p = <0.001	0.039<br>[4%]<br>p = 0.105	0.399<br>[49%]***<br>p = <0.001	1.946<br>[600.3%]***<br>p = <0.001	0.585<br>[79.5%]*<br>p = 0.039
4 years after	-0.015<br>[-1.5%]<br>p = 0.765	2.051<br>[677.8%]***<br>p = <0.001	-0.145<br>[-13.5%]<br>p = 0.642	-0.023<br>[-2.3%]<br>p = 0.285	0.524<br>[69%]***<br>p = <0.001	0.019<br>[1.9%]<br>p = 0.375	0.415<br>[51.4%]***<br>p = <0.001	2.038<br>[667.9%]***<br>p = <0.001	0.651<br>[91.7%]<br>p = 0.064
5 years after	-0.029<br>[-2.9%]<br>p = 0.607	2.111<br>[725.3%]**<br>p = 0.002	-0.174<br>[-16%]<br>p = 0.649	-0.027<br>[-2.7%]<br>p = 0.256	0.611<br>[84.3%]**<br>p = 0.002	0.018<br>[1.9%]<br>p = 0.498	0.43<br>[53.8%]***<br>p = <0.001	2.1<br>[716.7%]***<br>p = <0.001	0.698<br>[100.9%]<br>p = 0.094
6 years after or more	-0.043<br>[-4.2%]<br>p = 0.505	2.263<br>[861.3%]**<br>p = 0.003	-0.353<br>[-29.7%]<br>p = 0.456	-0.033<br>[-3.2%]<br>p = 0.218	0.674<br>[96.3%]**<br>p = 0.002	0.01<br>[1%]<br>p = 0.753	0.459<br>[58.2%]***<br>p = <0.001	2.168<br>[774.3%]**<br>p = 0.001	0.689<br>[99.1%]<br>p = 0.171
Num.Obs.	16377	675	397	16377	675	397	16225	664	384
R2	0.967	0.931	0.913	0.888	0.864	0.843	0.870	0.893	0.861
R2 Adj.	0.963	0.920	0.897	0.874	0.842	0.814	0.853	0.875	0.834
FE Bio.	1829	75	44	1829	75	44	1828	75	44
FE Years	9	9	9	9	9	9	9	9	9

Table SM17: Event study, with a 7-year time window. Estimated staggered effects (Two-Way Fixed Effects, TWFE) relative to the event's timing. The models incorporate individual and time-fixed effects. The percentual changes in the dependent variable are shown in brackets, calculated as $(e\widehat{(\beta_k)} - 1) \times 100$. Standard errors are clustered at the individual and year levels. Statistical significance levels: * p < 0.05, ** p < 0.01, *** p < 0.001.

**Dep.Var**		**Log(PageViews)**			**Log(Locality)**			**Log(Globality)**	
**Event**	**Death**	**Nobel**	**Oscar**	**Death**	**Nobel**	**Oscar**	**Death**	**Nobel**	**Oscar**
**Rel. Time**									
7 years before or more	-0.019<br>[-1.9%]<br>p = 0.422	-0.493<br>[-38.9%]*<br>p = 0.018	-0.787<br>[-54.5%]<br>p = 0.471	0.005<br>[0.5%]<br>p = 0.557	-0.439<br>[-35.5%]***<br>p = <0.001	0.074<br>[7.6%]<br>p = 0.696	-0.204<br>[-18.4%]***<br>p = <0.001	0.036<br>[3.7%]<br>p = 0.801	1.129<br>[209.3%]<br>p = 0.147
6 years before	0.004<br>[0.4%]<br>p = 0.786	-0.668<br>[-48.7%]*<br>p = 0.033	-0.525<br>[-40.8%]<br>p = 0.569	0.011<br>[1.1%]<br>p = 0.108	-0.479<br>[-38.1%]***<br>p = <0.001	0.037<br>[3.7%]<br>p = 0.785	-0.167<br>[-15.4%]**<br>p = 0.001	-0.196<br>[-17.8%]<br>p = 0.064	0.915<br>[149.8%]<br>p = 0.174
5 years before	-0.01<br>[-1%]<br>p = 0.712	-0.484<br>[-38.3%]**<br>p = 0.01	-0.617<br>[-46%]<br>p = 0.353	0.001<br>[0.1%]<br>p = 0.841	-0.36<br>[-30.2%]***<br>p = <0.001	0.033<br>[3.3%]<br>p = 0.777	-0.145<br>[-13.5%]***<br>p = <0.001	-0.269<br>[-23.6%]*<br>p = 0.03	0.8<br>[122.6%]<br>p = 0.147
4 years before	-0.021<br>[-2%]<br>p = 0.294	-0.289<br>[-25.1%]*<br>p = 0.016	-0.397<br>[-32.8%]<br>p = 0.463	-0.006<br>[-0.6%]<br>p = 0.368	-0.276<br>[-24.1%]***<br>p = <0.001	0.04<br>[4.1%]<br>p = 0.628	-0.119<br>[-11.3%]***<br>p = <0.001	-0.177<br>[-16.2%]<br>p = 0.31	0.488<br>[62.9%]<br>p = 0.215
3 years before	-0.025<br>[-2.4%]<br>p = 0.09	-0.221<br>[-19.8%]**<br>p = 0.002	-0.262<br>[-23.1%]<br>p = 0.468	-0.004<br>[-0.4%]<br>p = 0.66	-0.179<br>[-16.4%]***<br>p = <0.001	0.017<br>[1.7%]<br>p = 0.758	-0.077<br>[-7.4%]**<br>p = 0.001	-0.004<br>[-0.4%]<br>p = 0.975	0.435<br>[54.4%]<br>p = 0.089
2 years before	-0.005<br>[-0.5%]<br>p = 0.682	-0.029<br>[-2.8%]<br>p = 0.488	-0.136<br>[-12.7%]<br>p = 0.565	0.003<br>[0.3%]<br>p = 0.576	-0.053<br>[-5.1%]*<br>p = 0.047	0.029<br>[3%]<br>p = 0.498	-0.047<br>[-4.6%]**<br>p = 0.004	-0.032<br>[-3.2%]<br>p = 0.663	0.257<br>[29.3%]*<br>p = 0.025
**Event year**	**1.035<br>[181.4%]***<br>p = <0.001**	**2.734<br>[1439.6%]***<br>p = <0.001**	**0.554<br>[74%]*<br>p = 0.019**	**0.201<br>[22.3%]***<br>p = <0.001**	**0.645<br>[90.7%]***<br>p = <0.001**	**0.059<br>[6.1%]<br>p = 0.078**	**0.145<br>[15.6%]**<br>p = 0.002**	**1.237<br>[244.5%]***<br>p = <0.001**	**-0.28<br>[-24.4%]<br>p = 0.065**
1 years after	0.16<br>[17.3%]***<br>p = <0.001	2.251<br>[849.6%]***<br>p = <0.001	0.821<br>[127.4%]*<br>p = 0.047	0.016<br>[1.7%]*<br>p = 0.045	0.564<br>[75.8%]***<br>p = <0.001	0.077<br>[8%]<br>p = 0.194	0.399<br>[49.1%]***<br>p = <0.001	1.655<br>[423.4%]***<br>p = <0.001	-0.176<br>[-16.1%]<br>p = 0.54
2 years after	0.066<br>[6.9%]**<br>p = 0.002	2.028<br>[659.8%]***<br>p = <0.001	0.439<br>[55.2%]<br>p = 0.339	-0.001<br>[-0.1%]<br>p = 0.921	0.548<br>[72.9%]***<br>p = <0.001	0.005<br>[0.5%]<br>p = 0.95	0.452<br>[57.1%]***<br>p = <0.001	1.759<br>[480.8%]***<br>p = <0.001	0.033<br>[3.3%]<br>p = 0.937
3 years after	0.055<br>[5.6%]**<br>p = 0.003	1.924<br>[585%]***<br>p = <0.001	0.504<br>[65.6%]<br>p = 0.435	-0.008<br>[-0.8%]<br>p = 0.309	0.582<br>[79%]***<br>p = <0.001	-0.009<br>[-0.9%]<br>p = 0.926	0.497<br>[64.4%]***<br>p = <0.001	1.791<br>[499.6%]***<br>p = <0.001	-0.057<br>[-5.5%]<br>p = 0.904
4 years after	0.029<br>[2.9%]<br>p = 0.072	1.929<br>[588.4%]***<br>p = <0.001	0.371<br>[44.9%]<br>p = 0.631	-0.016<br>[-1.6%]<br>p = 0.253	0.608<br>[83.7%]***<br>p = <0.001	-0.042<br>[-4.1%]<br>p = 0.741	0.538<br>[71.2%]***<br>p = <0.001	1.845<br>[532.8%]***<br>p = <0.001	-0.15<br>[-13.9%]<br>p = 0.799
5 years after	0.023<br>[2.3%]<br>p = 0.074	1.964<br>[612.7%]***<br>p = <0.001	0.446<br>[56.2%]<br>p = 0.637	-0.019<br>[-1.9%]<br>p = 0.226	0.711<br>[103.7%]***<br>p = <0.001	-0.054<br>[-5.2%]<br>p = 0.73	0.578<br>[78.2%]***<br>p = <0.001	1.868<br>[547.5%]***<br>p = <0.001	-0.265<br>[-23.2%]<br>p = 0.71
6 years after	0.023<br>[2.4%]<br>p = 0.071	2.067<br>[689.8%]***<br>p = <0.001	0.385<br>[46.9%]<br>p = 0.725	-0.021<br>[-2.1%]<br>p = 0.313	0.777<br>[117.5%]***<br>p = <0.001	-0.078<br>[-7.5%]<br>p = 0.67	0.628<br>[87.4%]***<br>p = <0.001	1.868<br>[547.3%]***<br>p = <0.001	-0.388<br>[-32.2%]<br>p = 0.656
7 years after or more	0.012<br>[1.2%]<br>p = 0.411	2.084<br>[704%]***<br>p = <0.001	0.487<br>[62.8%]<br>p = 0.71	-0.028<br>[-2.8%]<br>p = 0.277	0.827<br>[128.6%]***<br>p = <0.001	-0.085<br>[-8.1%]<br>p = 0.696	0.661<br>[93.7%]***<br>p = <0.001	1.875<br>[552.3%]***<br>p = <0.001	-0.7<br>[-50.4%]<br>p = 0.518
Num.Obs.	16377	675	397	16377	675	397	16225	664	384
R2	0.967	0.931	0.914	0.888	0.865	0.844	0.870	0.893	0.862
R2 Adj.	0.963	0.920	0.897	0.874	0.842	0.813	0.853	0.875	0.834
FE Bio.	1829	75	44	1829	75	44	1828	75	44
FE Years	9	9	9	9	9	9	9	9	9

Temporal decay of collective memory

To examine how recognition of historical figures changes with time, we analyzed the relationship between the proportion of correct answers and the historical age of each figure (measured as years since birth)[2]. As expected, recognition declines over time. However, the shape and rate of this decline varies depending on whether the figure shares the respondent's country of origin.

Biographies from the same country follow a two-phase decay pattern (Figure SM11): an initial steep decline followed by a slower, more persistent phase. In contrast, biographies from different countries exhibit a smoother, single-phase decline. These patterns are consistent with the idea that same-country figures benefit from both communicative and cultural memory mechanisms, whereas different-country figures are remembered primarily through cultural channels.

To quantify these dynamics, we evaluated three models of memory decay:

Biexponential Decay (Communicative + Cultural Memory)

The biexponential model accounts for two distinct decay processes:

$$S(t) = N \cdot [\, e^{\{-(p+r)t\}} + \frac{r}{p+r-q} \cdot (e^{\{-qt\}} - e^{\{-(p+r)t\}})\,] \quad (6)$$

Where N is the initial level of recognition, p is the communicative memory decay rate, r is the transfer rate to cultural memory, q is the cultural memory decay rate. The model predicts a critical time t_c, defined as the time at which both memory processes contribute equally. It is given by:

$$t_c = \frac{1}{p+r-q} \cdot log(\frac{(p+r)(p-q)}{rq}) \quad (7)$$

For same-country figures, the model estimates an initial recognition level of N = 0.97, with slower communicative decay and transfer rates (p = 0.0139, r = 0.0181) and a lower cultural decay rate (q = 0.00038). For different-country figures, the initial recognition is lower (N = 0.61), and both the communicative decay and transfer rates are faster (p = 0.0147, r = 0.0356), along with a higher cultural decay rate (q = 0.00080). These parameter differences result in a longer persistence of

[2] Candia et al., "The Universal Decay of Collective Memory and Attention."

communicative memory for same-country figures as reflected in the estimated critical transition time: 131 years for same-country figures compared to 64 years for different-country figures (see Table SM18).

Exponential Decay (Single-Process Memory Loss)

We also evaluated a simple exponential model, which assumes recognition declines at a constant rate:

$$S(t) = c \cdot e^{\{-qt\}} \quad (8)$$

This model provides a baseline for comparison but lacks the capacity to capture dual memory phases. It yielded substantially poorer fits across both groups (Table SM19), consistent with the observed deviations from constant-rate decay in the empirical data.

Log-normal Modulated Power Law (Burst and Long-Tail Memory)

Lastly, we fit a log-normal model, commonly used to describe attention dynamics in online behavior and citation networks:

$$S(t) = e^{b} \cdot t^{b_1} \cdot e^{\left(-b_2 \cdot (\log t)^2\right)} \quad (9)$$

This model captures early bursts of attention followed by gradual decline. For different-country figures, the log-normal model yielded the best fit among all three alternatives (Table SM19), suggesting that their recognition follows a smoother, single-process decay dynamic. For same-country figures, however, the biexponential model provided a significantly better fit, supporting the presence of dual memory mechanisms in that group.

Together, the results indicate that shared national identity is associated with more persistent recognition across time. For same-country figures, this persistence reflects the joint influence of communicative and cultural memory mechanisms, which together produce a two-phase decay trajectory. For different-country figures, recognition appears to rely primarily on long-term cultural retention, consistent with the single-phase pattern captured by the log-normal model.

Toward the upper end of the age distribution (above 100 years), we observe a small uptick in the LOESS-smoothed accuracy curves, particularly for different-country figures. This is likely an artifact driven by data sparsity in the tail of the distribution, where relatively few figures remain. Supporting this, the histogram and cumulative distribution in Figure SM12 show that more than 60% of figures in the dataset are younger than 150 years. However, alternative explanations are worth noting: selection bias (where only extremely well-known older figures remain) and commemorative effects (e.g., centennials) may also contribute to this uptick. While interesting, these alternative explanations are speculative, and we interpret the trend with caution and attribute it primarily to smoothing artifacts caused by data sparsity.

Table SM18: Biexponential model parameters and model fit by country match.

Country	N	p	r	q	R^2	AIC	BIC	Tc	Sd (Tc)
Same	0.9681	0.0139	0.0181	0.00038	0.8780	–2006.27	–1986.63	131.49	17.10
Different	0.6147	0.0147	0.0356	0.00080	0.9038	–6487.54	–6463.08	64.73	4.55

Table SM19: Model fit comparison by model type and country match.

Country	Model	AIC	BIC
Same	biexponential	–2006.27	–1986.63
Same	lognormal	–1979.15	–1963.43
Same	exponential	–1562.47	–1550.69
Different	lognormal	–6834.27	–6814.70
Different	biexponential	–6487.54	–6463.08
Different	exponential	–5796.96	–5782.28

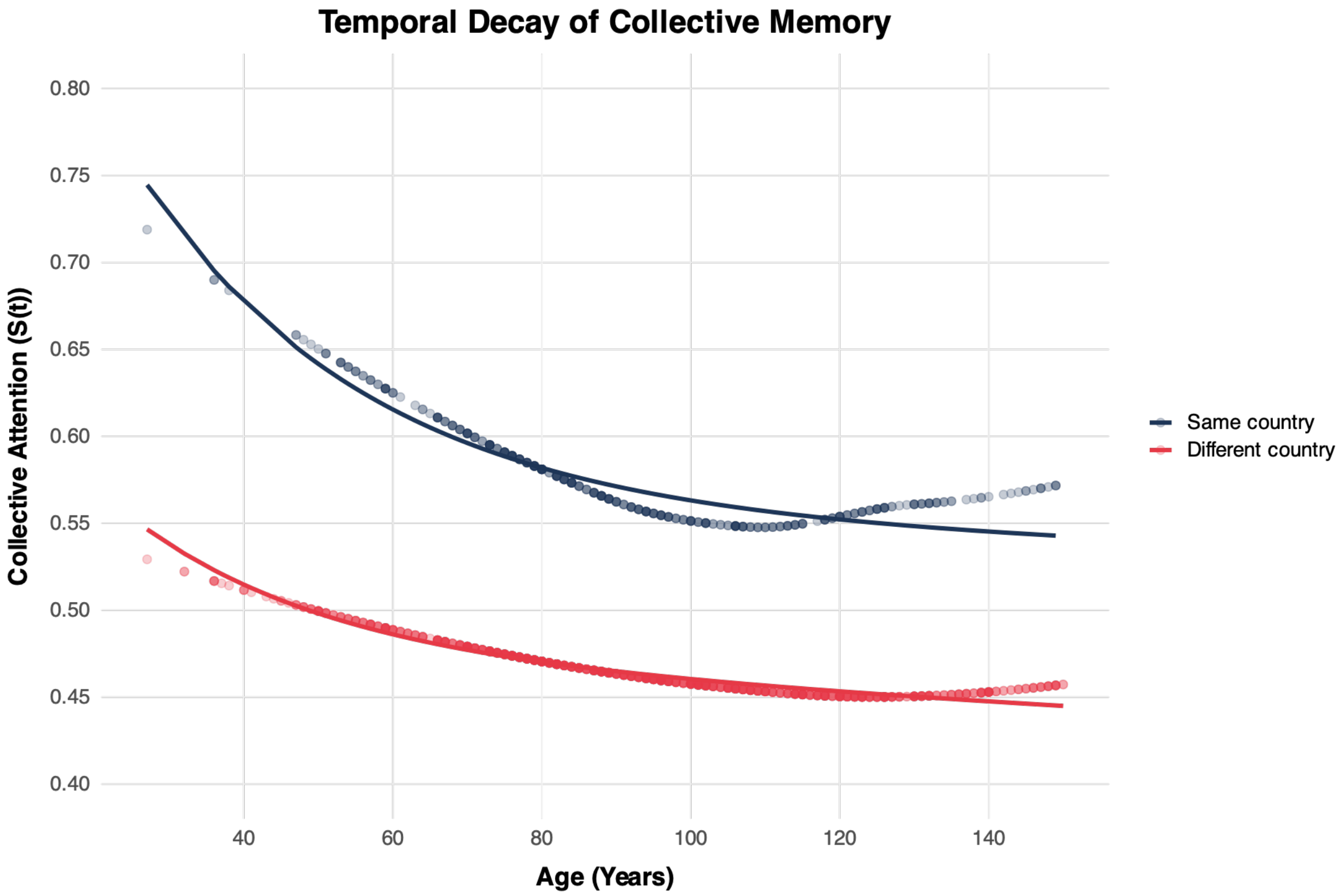

Figure SM11. Temporal decay in historical knowledge of cultural icons. *LOESS-smoothed average recognition accuracy by historical age. Same-country figures exhibit a two-phase decay pattern; different-country figures show a single-phase decline.*

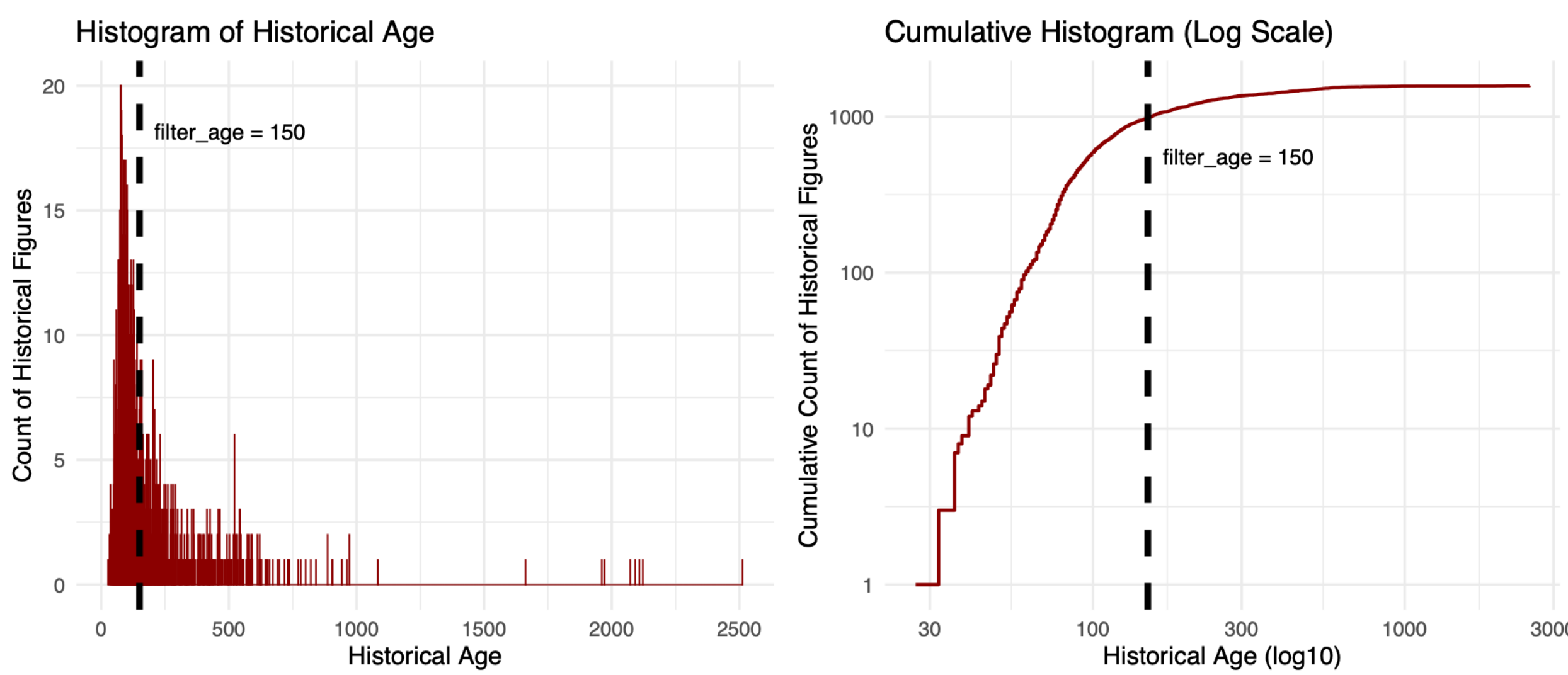

Figure SM12. Distribution of historical ages in the sample. *Histogram and cumulative distribution of historical ages show that most figures are less than 150 years old, highlighting sparsity in the tail.*
